# Ferrous Pyrophosphate Hollow Nanorods Enable Lithium Metal Batteries Under Low Negative/Positive Capacity Ratios

**DOI:** 10.1002/adma.74276

**Published:** 2026-07-28

**Authors:** Chen Yu, Jiarui Yang, Deyan Luan, Xiong Wen (David) Lou

**Affiliations:** ^1^ Department of Chemistry City University of Hong Kong Kowloon Hong Kong China

**Keywords:** lithium metal anodes, lithiophilic hosts, lithium metal batteries, low N/P capacity ratio, pyrophosphate

## Abstract

Lithium (Li) metal batteries (LMBs) exhibit a promising paradigm for their high energy density. However, their realization is still hindered by the inherent instability and excess of Li metal. Herein, we rationally design and synthesize ferrous pyrophosphate hollow nanorods encapsulated by an N‐doped carbon shell (Fe_2_P_2_O_7_@NC) as a Li host for Li metal anodes (LMAs). The Fe_2_P_2_O_7_@NC‐casted Cu electrode (Fe_2_P_2_O_7_@NC‐Cu) guides uniform Li deposition and suppresses volume expansion, enabling uniform Li plating and enhanced Coulombic efficiency. Time‐of‐flight secondary ion mass spectrometry and in‐depth X‐ray photoelectron spectroscopy analyses reveal that the existence of Fe_2_P_2_O_7_ promotes the generation of LiF and Li_2_O in LMAs, thereby promoting uniform Li plating. The Fe_2_P_2_O_7_@NC‐Li electrode presents stable Li deposition/stripping with small voltage polarization for 1400 h at 1 mA cm^−2^ and 1 mAh cm^−2^. Furthermore, a full cell coupled with a LiFePO_4_ cathode, demonstrates consistent cycling stability, maintaining a capacity retention of 80.6% over 136 cycles at a low negative/positive capacity ratio of 1.5, highlighting the great potential of Fe_2_P_2_O_7_@NC for practical high‐energy‐density LMBs.

## Graphical abstract

N‐doped carbon‐coated ferrous pyrophosphate (Fe_2_P_2_O_7_@NC) hollow nanorods are rationally synthesized through a self‐template method. The Fe_2_P_2_O_7_@NC hollow nanorods facilitate uniform Li deposition as a Li host by generating LiF on the electrode/electrolyte interface, and form Li_2_O in the bulk phase of the electrode. The obtained full cell using a LiFePO_4_ cathode achieves stable cycling performance under low negative/positive capacity ratios.

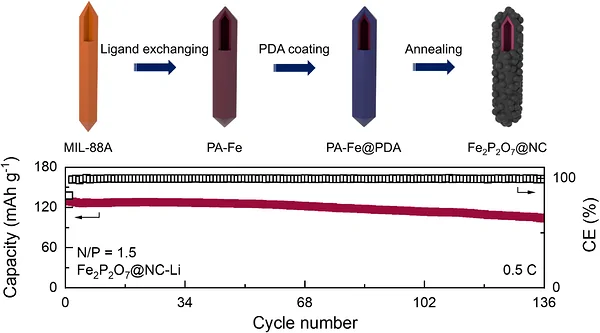

## 1 Introduction

Although Li‐ion batteries (LIBs) have been commercialized for over 30 years, their low energy density is anticipated to pose challenges in meeting the demands of future industrial development. Li metal batteries (LMBs), which employ Li metal as an anode, are regarded as attractive next‐generation electrochemical energy storage devices. LMBs can fundamentally overcome the limitations of LIBs for the high theoretical capacity (∼3860 mAh g^−1^) and ultra‐low theoretical reduction potential (−3.04 V vs. standard hydrogen electrode) of Li metal, leading to a superior theoretical energy density of LMBs [1, 2]. However, practical application of LMBs faces a sequence of inherent challenges by unstable Li metal anodes (LMAs), including uneven Li metal deposition and uncontrollable side reactions on Li/electrolyte interface. These issues result in undesirable Coulombic efficiency (CE) and deficient cycling performance [3, 4]. To overcome the above issues, numerous strategies have been explored, including constructing 3D Li hosts [5, 6], fabricating an artificial solid electrolyte interface (SEI) layer [7, 8], electrolyte engineering [9, 10], separator modification [1, 11], etc. Among these approaches, designing 3D Li hosts has been proven effective for stabilizing LMAs, as they homogenize Li deposition and confine Li metal within their 3D structures, thereby enabling long‐lifespan LMBs [4, 12]. Various lithiophilic species, such as metals [5, 13] and metal oxides [14, 15], are synthesized and employed to guide uniform Li deposition and achieve prolonged cycle life of LMBs. Polyanionic materials have been of recent interest as cathode materials for sodium batteries [16, 17], potassium batteries [18], etc., due to their unique structural and physicochemical properties [19, 20]. Among them, pyrophosphates, such as Na_2_FeP_2_O_7_ and Zn_2_P_2_O_7_, the featured P_2_O_7_^4−^ polyhedral units construct stable 3D frameworks effective in mitigating volume changes during charge and discharge processes [21, 22]. Substantial volume change in the anode poses a significant challenge in LMBs, and investigation of pyrophosphates in this realm remains uncommon.

In addition to the above issues, most current studies employ LMAs with much excess of Li metal to realize extended cycle life of LMBs [23, 24]. The excess Li metal has the ability to compensate Li^+^ ion loss in cathode material during battery cycling [25]. Indeed, the surplus Li metal does not give any contribution to the energy density of the LMBs, but rather erodes the advantages in energy density of LMBs over LIBs, escalating manufacturing costs and compromising storage and operational safety [26]. The negative/positive capacity (N/P) ratio is a critical parameter that qualifies the excess rate of anode capacity. Developing LMBs under low N/P ratios (i.e., minimal anode Li excess) is urgently needed to fully exploit the ultra‐high energy density feature of LMBs [27]. However, achieving stable cycling under such harsh conditions remains a significant challenge.

Herein, Fe_2_P_2_O_7_ hollow nanorods encapsulated by N‐doped carbon (Fe_2_P_2_O_7_@NC) are synthesized and cast on bare Cu foil (b‐Cu) to form electrodes with a unique Li host (Fe_2_P_2_O_7_@NC‐Cu) to stabilize LMAs and realize high‐performance LMBs under low N/P ratios. The presence of Fe_2_P_2_O_7_ promotes the formation of LiF on the electrode/electrolyte interface and Li_2_O in the bulk phase, which effectively facilitates uniform Li deposition. Besides, Fe_2_P_2_O_7_@NC also mitigates volume expansion during the Li deposition process. Therefore, the SEI layer is stabilized, and side reactions on Li/electrolyte interface are also suppressed, which are critical factors for maintaining stable CE during cell cycling. When coupled with a LiFePO_4_ (LFP) cathode at a current density of 0.5 C under a low N/P ratio of 1.5, the Fe_2_P_2_O_7_@NC‐Li anode demonstrates consistent cycling stability of 136 cycles. The feasibility of pairing the Fe_2_P_2_O_7_@NC‐Li anode with a high‑voltage NCM811 cathode has also been verified. The above advantages prove the promising potential of Fe_2_P_2_O_7_@NC for practical high‐energy‐density LMBs. Meanwhile, the pyrophosphates discussed in this work offer a new material platform for the construction of effective Li hosts.

## 2 Results and Discussion

### 2.1 Synthesis and Characterization of Fe_2_P_2_O_7_@NC Hollow Nanorods

**FIGURE 1:**
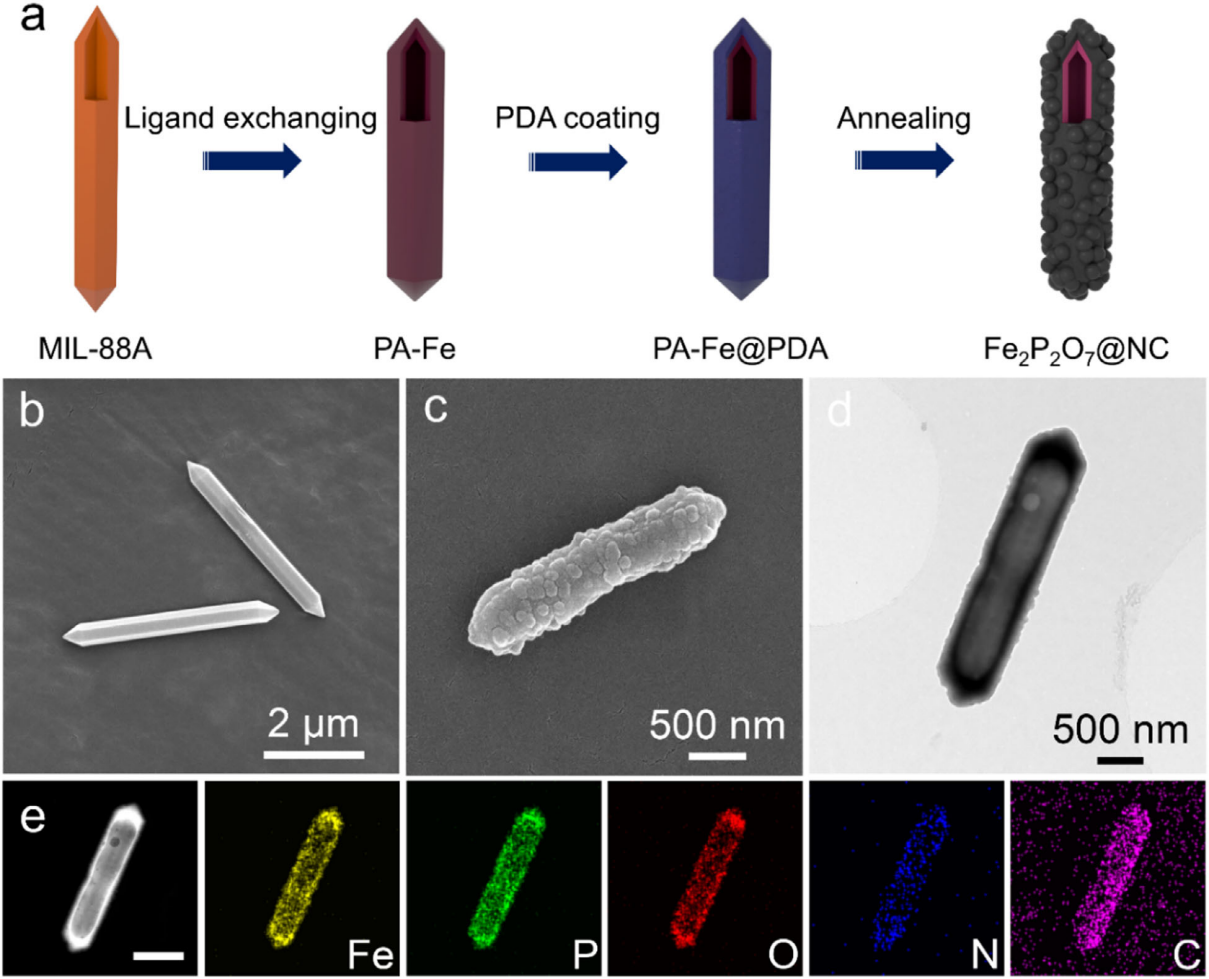
Formation process of Fe_2_P_2_O_7_@NC. (a) Schematic diagram of the synthesis of Fe_2_P_2_O_7_ @NC nanorods. FESEM images of (b) MIL‐88A and (c) Fe_2_P_2_O_7_@NC. (d) TEM image and (e) HAADF‐STEM and elemental mapping images of Fe, P, O, N, and C elements of a single Fe_2_P_2_O_7_@NC nanorod, scale bar is 1 µm.

The synthesis of Fe_2_P_2_O_7_@NC hollow nanorods begins with hexagonal Materials of the Institute Lavoisier‐88A (MIL‐88A) nanorods (Figure 1a) by a self‐template method [28]. The field‐emission scanning electron microscopy (FESEM) image reveals that the MIL‐88A nanorods exhibit an average length and width of 4.2 µm and 440 nm, respectively (Figure 1b). Then, the MIL‐88A nanorods are etched by 0.2 M phytic acid (PA) solution to obtain quasi‐hexagonal PA‐Fe hollow nanorods (Figure S1). During this step, the ligand of MIL‐88A is fully replaced by PA molecules from the original fumaric acid ligand, as confirmed by comparing the X‐ray diffraction (XRD) patterns of MIL‐88A and PA‐Fe (Figures S2a and S3a) [28]. Additionally, in comparison to MIL‐88A, the existence of P element in the energy‐dispersive X‐ray spectrum (EDX) result of PA‐Fe further proves the introduction of PA ligand (Figures S2b and S3b). Subsequently, the PA‐Fe hollow nanorods are coated by polydopamine layer to obtain PA‐Fe@PDA hollow nanorods via in situ polymerization of dopamine molecules in Tris‐buffer aqueous solution (Figure S4), the product is subsequently converted into Fe_2_P_2_O_7_@NC hollow nanorods via the carbonization process.

The FESEM images of as‐obtained Fe_2_P_2_O_7_@NC reveal a rod‐like morphology (Figure 1c; Figure S5), and the transmission electron microscopy (TEM) image indicates that the nanorod retains a similar hollow structure of PA‐Fe@PDA (Figure 1d). The high‐angle annular dark‐field scanning transmission electron microscope (HAADF‐STEM) image of Fe_2_P_2_O_7_@NC further confirms its hollow structure, and elemental mapping images showcase the Fe, P, O, N, and C elements homogeneously distributed in Fe_2_P_2_O_7_@NC hollow nanorods (Figure 1e). Besides, the XRD pattern (Figure S6a) of Fe_2_P_2_O_7_@NC exhibits clear signals of Fe_2_P_2_O_7_, the EDX spectrum and EDX line scanning results also confirm the existence of Fe, P, O, N, and C elements (Figure S6b and S7). Moreover, the Raman spectrum also exhibits two notable peaks attributed to G band (sp^2^ carbon) and D band (disordered carbon) of carbon, respectively (Figure S8) [29, 30]. The valence state and chemical composition of Fe_2_P_2_O_7_@NC are studied by X‐ray photoelectron spectroscopy (XPS) measurements, and the signals of Fe, P, O, N, and C elements are clearly detected (Figure S9). In the high‑resolution Fe 2p spectrum, the peaks at 711.0 and 723.9 eV are assigned to Fe^2+^, while the peaks at 712.8 and 725.7 eV are attribute to Fe^3+^. The peaks at 714.7 and 728.3 eV correspond to satellite peaks (denoted as sat) of Fe^2+^, and another two peaks at 718.8 eV and 733.5 eV are attributed to satellite peaks of Fe^3+^ [31]. The existence of Fe^3+^ is possibly due to the slight oxidation of the sample surface [32]. The peak at 133.7 eV in high‐resolution P 2p spectrum is attributed to the existence of PO_4_^3−^ [33]. In the high‐resolution O 1s spectrum, the peaks at 533.7 and 531.9 eV are assigned to O─C═O and P─O, respectively [34, 35]. These results are consistent with the XRD result of the sample. In the high‐resolution C 1s spectrum, the three deconvoluted peaks located at 284.8, 286.3, and 288.7 eV are assigned to C═C/C─C, C─N, and O─C═O, respectively [36, 37]. Besides, there are two peaks (398.0 and 400.0 eV) in the high‐resolution N 1s spectrum, which are attributed to pyridinic‐N and graphitic‐N [38, 39, 40].

### 2.2 Li Deposition Behavior Investigation

**FIGURE 2:**
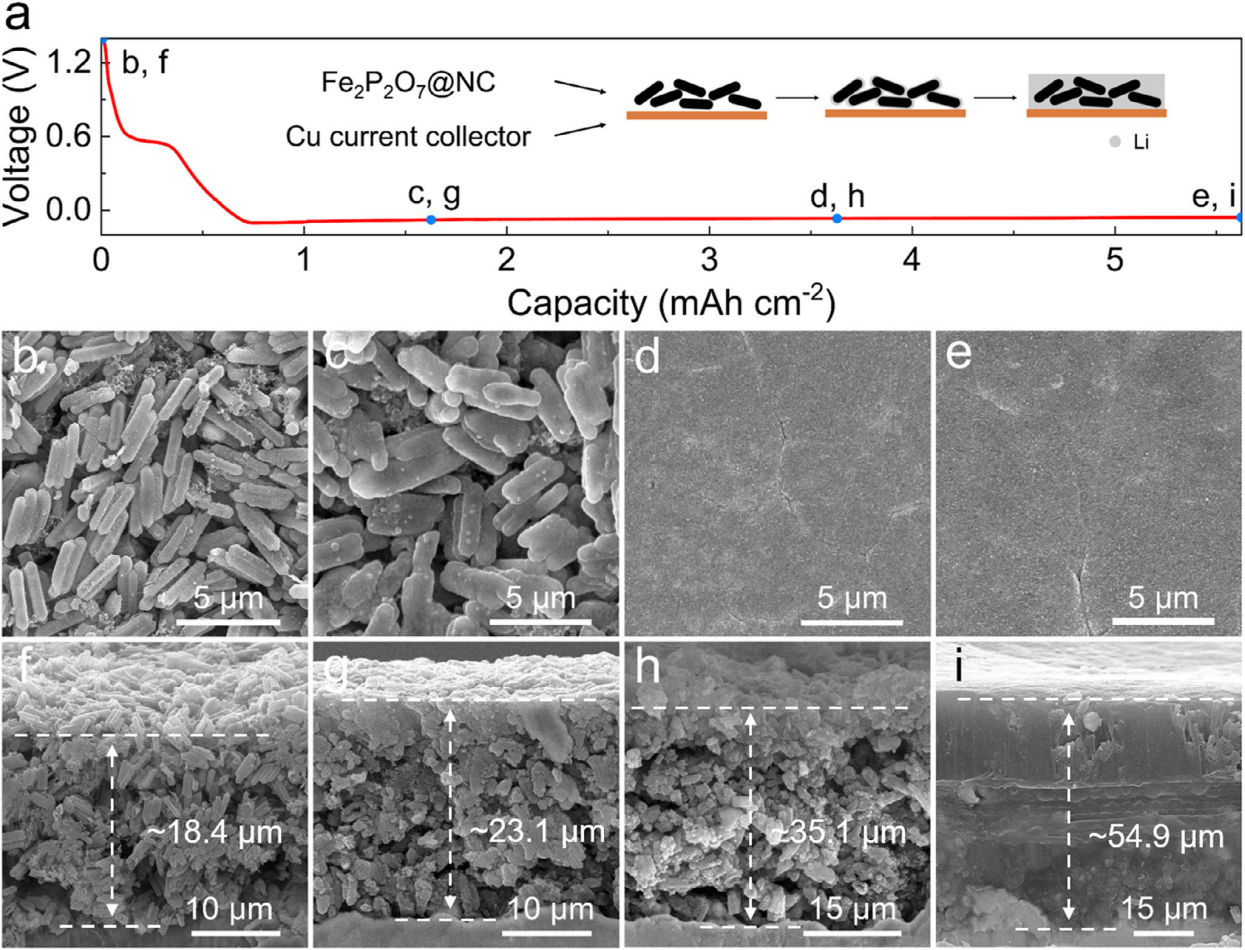
Li deposition behavior on the Fe_2_P_2_O_7_@NC‐Cu. (a) Discharge curve of Fe_2_P_2_O_7_@NC‐Cu//Li half cell and schematic diagram during the Li deposition process on the Fe_2_P_2_O_7_@NC‐Cu electrode. (b) Top and (f) cross‐sectional FESEM images of a fresh Fe_2_P_2_O_7_@NC‐Cu electrode, (c–e) Top and (g‐i) cross‐sectional FESEM images of Li deposited with capacities of (c,g) 1 mAh cm^−2^, (d,h) 3 mAh cm^−2^, and (e,i) 5 mAh cm^−2^.

The Li deposition behavior influences the cycling performance of batteries. Uncontrolled Li deposition on the anode/electrolyte interface triggers detrimental issues, including Li dendrite growth, aggravated side reactions, dead Li formation, and an unstable SEI layer, leading to battery failure and safety issues [41, 42, 43]. To study Li deposition behavior, various areal capacities of metallic Li are deposited on Fe_2_P_2_O_7_@NC‐Cu and b‐Cu electrodes at 1 mA cm^−2^. The representative discharge curve of Fe_2_P_2_O_7_@NC‐Cu//Li half cell is presented (Figure 2a). Compared to the fresh electrode shown in Figure 2b, Li deposition mainly occurs on the surface of nanorods as the Li deposition capacity reaches 1 mAh cm^−2^ (Figure 2c). With a Li deposition capacity of 3 mAh cm^−2^, a flat layer of Li metal forms at the interface of the electrode without any obvious Li dendrites (Figure 2d). With 5 mAh cm^−2^ of Li deposited, FESEM analysis reveals complete filling of the electrode's internal voids with Li metal, accompanied by flat surface Li plating, and no obvious Li dendrites are identified (Figure 2e,i). In contrast, Li deposition on the b‐Cu electrode exhibits noticeable unevenness (Figure S10).

Furthermore, the cross‐sectional FESEM images of Fe_2_P_2_O_7_@NC‐Cu (Figure 2g–i) illustrate that the thickness of Fe_2_P_2_O_7_@NC‐Cu increases by 4.7, 16.7, and 36.5 µm at Li plating capacities of 1, 3, and 5 mAh cm^−2^, respectively, compared to the fresh electrode (Figure 2f), indicating the superior ability of Fe_2_P_2_O_7_@NC to mitigate volume change, compared to b‐Cu (Figure S10). The top‐view and cross‐sectional FESEM observations illustrate that the spatial‐temporal sequence of Li deposition in the Fe_2_P_2_O_7_@NC‐Cu electrode entails concurrent Li deposition on the electrode/electrolyte interface and bulk phase. With shorter Li^+^ transport pathways, Li deposition occurs more swiftly at the electrode/electrolyte interface, followed by the gradual filling of void spaces in the porous bulk with metallic Li. Besides, the in situ optical microscopy results demonstrate that even after discharging for 90 min at a current density of 3 mA cm^−2^, the Fe_2_P_2_O_7_@NC‐Cu electrode shows uniform Li deposition without visible Li dendrites and minimal thickness expansion (Figure S11). Conversely, under identical testing conditions, the b‐Cu electrode rapidly develops Li dendrites that grow larger with extended discharge time, and exhibits a noticeable increase in electrode thickness (Figure S12). These observations together with the aforementioned FESEM observations of Li deposition highlight that the Fe_2_P_2_O_7_@NC enables flat and dendrite‐free Li deposition while mitigating volume expansion during Li deposition, suggesting the great potential of Fe_2_P_2_O_7_@NC as a high‐performance Li host for long‐lifespan LMBs.

### 2.3 Electrochemical Performance

**FIGURE 3:**
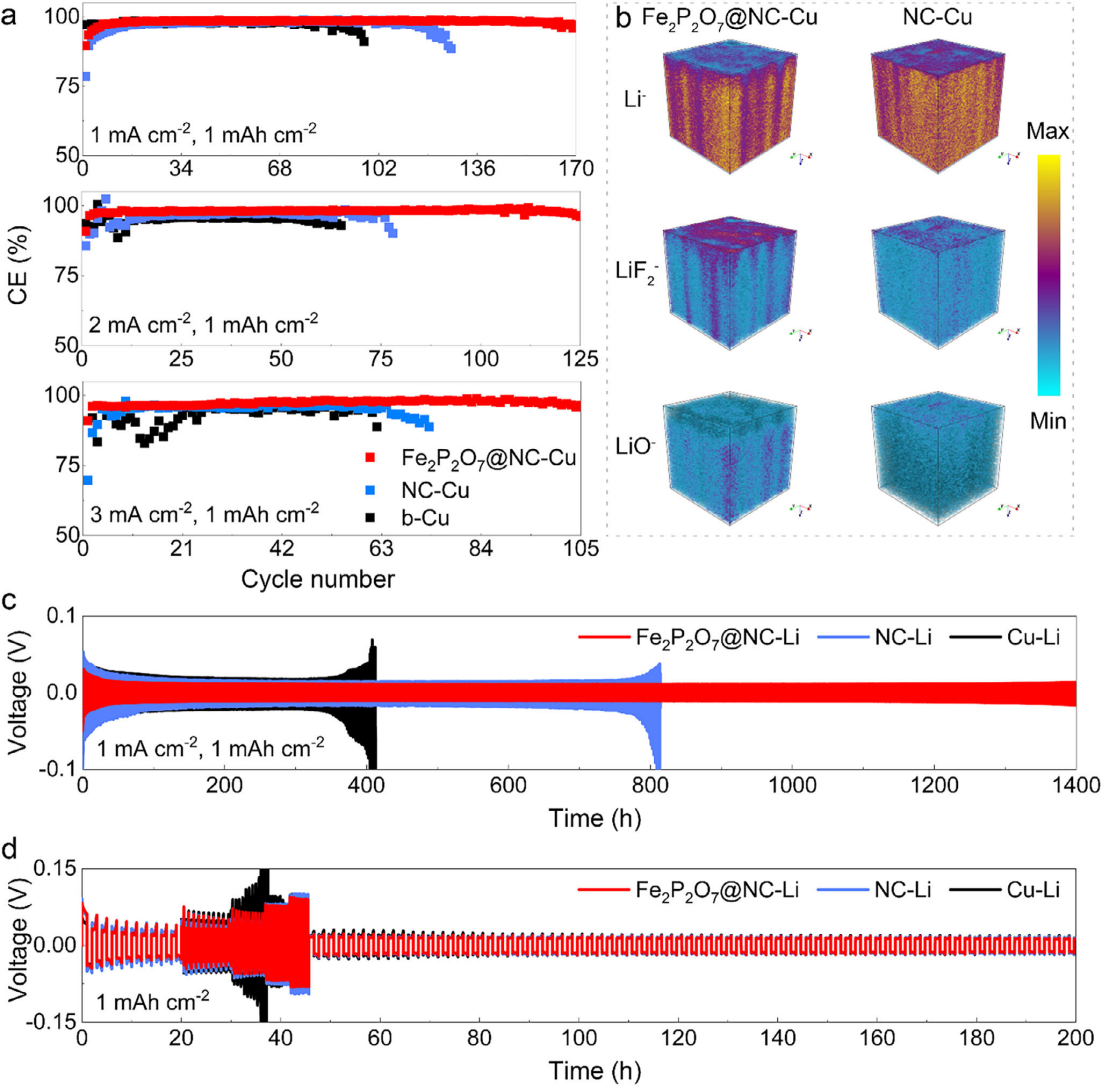
Electrochemical performance of different electrodes. (a) CE comparison of different electrodes at various current densities with a constant capacity of 1 mAh cm^−2^. (b) TOF‐SIMS investigation of Fe_2_P_2_O_7_@NC‐Cu and NC‐Cu electrodes after 100 cycles at 1 mA cm^−2^ and 1 mAh cm^−2^. (c) Galvanostatic cycling voltage profiles and (d) rate performance at varied current densities (1, 2, 3, 4, 5, and 1 mA cm^−2^) of different electrodes in symmetric cells with a constant capacity of 1 mAh cm^−2^.

CE is a key parameter for assessing the reversibility of Li deposition/stripping process, defined as the charge quantity ratio of Li stripping to Li deposition [44, 45]. To verify the stabilizing effect of Fe_2_P_2_O_7_ in Fe_2_P_2_O_7_@NC, we prepared a Fe_2_P_2_O_7_‐free NC sample as a control sample to isolate the contribution of NC in electrochemical performance (Figure S13). At 0.5 mA cm^−2^, the Fe_2_P_2_O_7_@NC‐Cu//Li cell achieves an average CE of 98.9% over the initial 200 cycles (Figure S14). The average CE of Fe_2_P_2_O_7_@NC‐Cu//Li cell maintains 98.31% at 1 mA cm^−2^ during the initial 170 cycles, while the NC‐Cu//Li cell experienced a CE decay after 115 cycles, and the b‐Cu//Li cell exhibits significant CE decay after only 90 cycles (Figure 3a). Besides, the voltage of the Fe_2_P_2_O_7_@NC‐Cu//Li cell shows a stable periodic variation over time, during the repeated Li deposition/stripping process (Figure S15). Furthermore, at 2 and 3 mA cm^−2^, the Fe_2_P_2_O_7_@NC‐Cu//Li cells also show better cycling stability, compared with the NC‐Cu//Li cell and b‐Cu//Li cell (Figure 3a). To investigate the reasons for the CE stability of Fe_2_P_2_O_7_@NC‐Cu//Li cells, voltage‐time profiles at 0.5 mA cm^−2^ of half cells with different electrodes are acquired (Figure S16). The result indicates that the Fe_2_P_2_O_7_@NC‐Cu electrode exhibits the lowest Li nucleation overpotential (28.1 mV) and the highest voltage plateau, suggesting its high lithiophilicity, superior interfacial Li^+^ transport and mass transfer capability, that optimize the even Li deposition on the electrode/electrolyte interface [3, 46]. Moreover, pre‐lithiation is performed on the Fe_2_P_2_O_7_@NC‐Cu//Li cell, followed by conducting in situ Fourier Transform Infrared spectroscopy measurements during the Li stripping process (Figure S17). The result reveals that there is no notable change of the characteristic peaks of the functional groups of the electrolyte (1058 cm^−1^ for C─O group, 1182 cm^−1^ for C‐F group, 1334 cm^−1^ for S═O group) during the Li stripping process. This result indicates that the side reactions on the Fe_2_P_2_O_7_@NC‐Li/electrolyte interface are effectively suppressed during the Li stripping process, thus, the irreversible Li loss during Li stripping is inhibited, thereby cycling reversibility of the cell is enhanced [47]. Meanwhile, combined with the above examination of cross‐sectional FESEM images of different substrates under different Li areal capacities (Figure 2b–i; Figure S10), the severe volume expansion by Li plating on b‐Cu can destabilize the SEI layer. The unstable SEI layer tends to rupture during cycling, which exacerbates the side reactions on the Li/electrolyte interface. The above process simultaneously induces the dendrites and dead Li formation, resulting in rapid decay of CE [48].

Moreover, we also conducted Time‐of‐flight secondary ion mass spectrometry (TOF‐SIMS) testing on the post‐cycling electrodes, which experienced 100 cycles in half cells for profiling the depth‐dependent chemical composition of the electrodes. The detected Li^−^ signal is typically associated with Li metal [49], suggesting that the presence of Li metal in the post‐cycling Fe_2_P_2_O_7_@NC‐Cu electrode is less than the post‐cycling NC‐Cu electrode, further confirming its superior retention of CE (Figure 3b; Figure S18). Additionally, the LiF_2_^−^ signal often corresponds to LiF species in the SEI layer. In comparison to the post‐cycling NC electrode, the signal of LiF_2_^−^ in the cycled Fe_2_P_2_O_7_@NC‐Cu electrode exhibits noticeable concentration on both the top surface and bulk phase (Figure 3b; Figure S19), indicating that the Fe_2_P_2_O_7_@NC‐Cu electrode promotes the decomposition of LiTFSI and generates LiF [50, 51, 52], a beneficial SEI component, which can shield the interior of the LMA while benefiting Li^+^ diffusion, improving the uniformity of Li deposition [53, 54, 55]. The TOF‐SIMS negative ion mapping of LiF_2_^−^ also indicates that the LiF distribution on the top surface of the cycled Fe_2_P_2_O_7_@NC‐Cu electrode is more concentrated compared with the post‐cycling NC‐Cu electrode (Figure S20). Meanwhile, Li_2_F_3_^−^ is considered a fragment signal of LiF as well [56], and its distribution in both electrodes further supports the above analysis of LiF distribution in the electrodes (Figures S21–S23). Moreover, the LiO^−^ signal is typically linked to the characteristic signal of Li_2_O [57]. The results reveal a more pronounced distribution of the LiO^−^ signal in the Fe_2_P_2_O_7_@NC‐Cu sample, while there is only a trace amount collected in the NC‐Cu sample (Figure 3b; Figure S24). Many studies have highlighted that Li_2_O is also a beneficial component of the SEI as well, offering favorable Li^+^ transport properties to the electrode and supporting the stable deposition of Li in LMAs [58, 59, 60]. The abundant Li_2_O in the bulk facilitates Li deposition within the electrode, which may explain why the Fe_2_P_2_O_7_@NC‐Cu electrode mitigates volume expansion during Li deposition. In addition to the TOF‐SIMS investigation discussed above, we also performed in‐depth XPS analysis on the post‐cycling Fe_2_P_2_O_7_@NC‐Cu electrode by Ar^+^ sputtering (Figure S25). The F 1s and Li 1s spectra reveal that while the surface of the electrode shows low LiF content, there is a notable LiF content in the superficial layers which decreases with depth [61, 62, 63]. Moreover, both the Li 1s and O 1s spectra provide evidence for the presence of LiF and Li_2_O in the electrode [64, 65, 66, 67].

We also examined the stability of the structure and chemical composition of Fe_2_P_2_O_7_@NC nanorods after undergoing 100 cycles at 2 mA cm^−2^ and 1 mAh cm^−2^ in a half cell (Figure S26). The TEM and FESEM images of the post‐cycling Fe_2_P_2_O_7_@NC reveal that the structure of hollow nanorods is well preserved, showcasing the exceptional structural stability of Fe_2_P_2_O_7_@NC for promoting uniform Li deposition over a long cycling process (Figure S26a,b). Moreover, the elemental mapping images acquired via FESEM‐EDX demonstrate that the elements remain evenly distributed within the nanorods, suggesting that the chemical composition of Fe_2_P_2_O_7_@NC remains stable throughout the cycling process (Figure S26c–h).

Symmetric cells are assembled by Li pre‐plated electrodes (Fe_2_P_2_O_7_@NC‐Li, NC‐Li and Cu‐Li) to evaluate Li^+^ ion transport ability and interfacial stability of the electrodes [68, 69]. As illustrated in Figure 3c, the Fe_2_P_2_O_7_@NC‐Li//Fe_2_P_2_O_7_@NC‐Li cell exhibits exceptional cycling stability for 1400 h at 1 mA cm^−2^ and 1 mAh cm^−2^ with ultra‐small voltage polarization (<23 mV, details are illustrated in Figure S27). While the voltage polarization of Cu‐Li//Cu‐Li and NC‐Li//NC‐Li cells begins to increase after over 400 h and 750 h (Figure S28) of cycling, respectively. Subsequently, rate performance tests of the symmetric cell are carried out by incrementally boosting the current density from 1 to 5 mA cm^−2^ (Figure 3d). The Fe_2_P_2_O_7_@NC‐Li//Fe_2_P_2_O_7_@NC‐Li cell exhibits voltage polarizations of 73.7 mV, 98.0 mV, 118.9 mV, 146.5 mV, and 171.1 mV, demonstrating robust cycling across all tested current densities. While the voltage polarization of the Cu‐Li//Cu‐Li cell increases rapidly after the current density rises to 3 mA cm^−2^. After increasing the current density to 5 mA cm^−2^, the cell initially cycles with abnormally high voltage polarization before exhibiting an abrupt drop in polarization voltage within a few cycles, which may be due to a soft short circuit in the cell [70]. The above results further underscore the exceptional Li^+^ ion transport capability of Fe_2_P_2_O_7_@NC and its effective regulation of Li deposition/stripping process.

**FIGURE 4:**
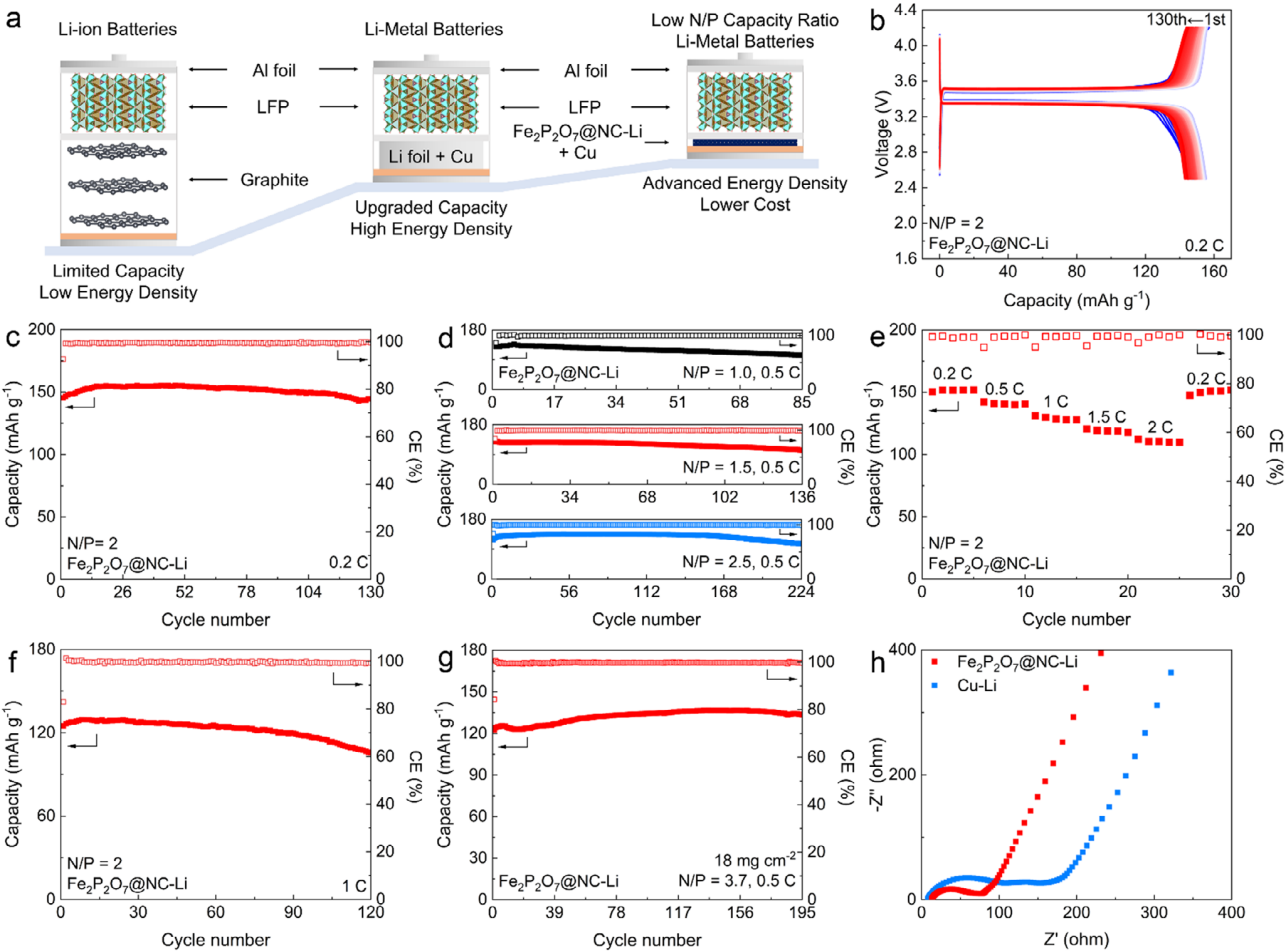
Electrochemical performance of full cells. (a) Schematic illustration of the excellence of low N/P ratio LMBs. (b) Charge/discharge curves and (c) cycling performance of Fe_2_P_2_O_7_@NC‐Li//LFP cell tested at 0.2 C (N/P = 2). (d) Cycling performance of Fe_2_P_2_O_7_@NC‐Li//LFP cell tested at different N/P ratios. (e) Rate capability of Fe_2_P_2_O_7_@NC‐Li//LFP cell tested at different current densities. (f, g) Cycling performance of Fe_2_P_2_O_7_@NC‐Li//LFP cell tested at (f) a high current density of 1 C and (g) a high mass loading of the cathode (18 mg cm^−2^) at 0.5 C. (h) Nyquist plots of Fe_2_P_2_O_7_@NC‐Li//LFP and Cu‐Li//LFP cells after cycling.

To verify the application potential of Fe_2_P_2_O_7_@NC hollow nanorods, full cells under low N/P ratios with Fe_2_P_2_O_7_@NC‐Li anodes and commercial LFP cathodes are assembled. Full cells using the same cathodes paired with NC‐Li and Cu‐Li anodes are also assembled for comparison. LFP is recognized as an attractive cathode material for future commercial LMBs for offering distinct advantages, including low cost, robust and stable charge/discharge voltage plateau, and high safety index [71, 72]. FESEM images, XRD pattern, EDX mapping images, and EDX spectrum of LFP powder are exhibited in Figures S29 and S30. Figure 4a illustrates the configuration of a low N/P ratio LMB and highlights its unique advantages in energy density and cost, compared with the commercialized LIBs and general LMBs. The cyclic voltammetry curves of the Fe_2_P_2_O_7_@NC‐Li//LFP cell overlap significantly, demonstrating the outstanding cycle reversibility of the cell (Figure S31). The charge/discharge curves of the Fe_2_P_2_O_7_@NC‐Li//LFP cell show an outstanding initial CE of 92.58% and stable cycling performance at 0.2 C (Figure 4b). Specifically, the cell retains 99.94% of its initial capacity over 130 cycles (Figure 4c). Under the same conditions, the NC‐Li//LFP cell exhibits faster capacity decay during cycling (Figure S32). Moreover, the cycling stability of Fe_2_P_2_O_7_@NC‐Li//LFP cells under different N/P ratios is assessed at 0.5 C (Figure 4d; Figure S33). Under an N/P ratio of 1.0, the cell maintains a capacity retention of 80.1% over 85 cycles. Increasing the N/P ratio to 2.5, the cell remains 89.2% of its initial capacity over 224 cycles (Figure 4d). The capacity retention of the full cells significantly improves as the N/P ratio increases. Under a higher N/P ratio of 6.2, the Fe_2_P_2_O_7_@NC‐Li//LFP cell also reaches a capacity retention of 95.8% over 515 cycles (Figure S34). The enhanced cycling performance originates from the abundant Li reservoir in the anode, which effectively compensate Li^+^ ion loss in the cathode material [73]. Besides, under a N/P ratio of 2, the Fe_2_P_2_O_7_@NC‐Li//LFP cell demonstrates superior rate capability compared to NC‐Li//LFP cell, maintaining robust capacity retention across multiple current densities (Figure 4e; Figure S35). Furthermore, the Fe_2_P_2_O_7_@NC‐Li//LFP cell exhibits outstanding cycling stability at an increased current density of 1 C, while NC‐Li//LFP cell experienced a rapid capacity decay under the same testing condition within 42 cycles (Figure 4f; Figures S36 and S37). More importantly, a high cathode mass loading is also a key factor towards high‐energy‐density LMBs. Figure S38 exhibits the cross‐sectional FESEM images of a high‐mass‐loading LFP cathode (18 mg cm^−2^). Equipping such a high‐mass‐loading LFP cathode, a highly stable cycling performance with an average CE of 99.66% without significant capacity decay after 195 cycles is achieved for the Fe_2_P_2_O_7_@NC‐Li//LFP cell (Figure 4g; Figure S39). As the N/P ratio of Fe_2_P_2_O_7_@NC‐Li//LFP cell with such a high LFP mass loading was reduced to 2, the cell also realized stable cycling for 184 cycles at 0.5 C, with a capacity loss rate of 0.108% in each cycle (Figure S40). Moreover, as the LFP mass loading was further increased to 21 mg cm^−2^ with a N/P ratio of 3.7, the Fe_2_P_2_O_7_@NC‐Li//LFP cell achieved a high capacity retention of 109.7% after 145 cycles (Figure S41). The high‐cathode‐loading full cells initially exhibit a capacity increase during cycling, which may be due to the progressive electrochemical activation of inner regions within the thick, high‐mass‐loading LFP cathodes [74]. The exceptional capacity retention of the full cell with high LFP mass loading also confirms its viability for application in high‐energy‐density LMBs. Electrochemical impedance spectroscopy results reveal that the Fe_2_P_2_O_7_@NC‐Li//LFP cell features a significantly lower charge transfer resistance compared with the Cu‐Li//LFP cell (Figure 4h), unveiling the improvement of reaction kinetics enabled by the Fe_2_P_2_O_7_@NC‐Li anode, thereby improving the capacity retention capability of the Fe_2_P_2_O_7_@NC‐Li//LFP cell [75]. The above full cell testing validates the feasibility of Fe_2_P_2_O_7_@NC‐Li//LFP cell under relatively rigorous LMBs configurations compared to other recent reports (Figure S42), and the cycling performance of the Fe_2_P_2_O_7_@NC‐Li anode is comparable to other previously reported Li anodes in full cells with LFP cathodes (Table S1). As discussed above, the impressive electrochemical features of the Fe_2_P_2_O_7_@NC‐Li//LFP cells under low N/P ratios have demonstrated its application potential in practical LMBs. Furthermore, we also evaluated the cycling performance of a full cell pairing the Fe_2_P_2_O_7_@NC‑Li anode with a NCM811 cathode, and the result demonstrates that its stable cycling is likewise achievable at a current density of 1 C (Figures S43–S46), suggesting the application of the Fe_2_P_2_O_7_@NC‑Li anode in LMBs can be further broadened.

## 3 Conclusions

In summary, Fe_2_P_2_O_7_ hollow nanorods encapsulated by N‐doped carbon shell are synthesized as a Li host for realizing LMBs under low N/P ratios. The Fe_2_P_2_O_7_@NC mitigates volume expansion by Li deposition, promotes LiF and Li_2_O formation on the electrode/electrolyte interface and bulk phase of electrode, respectively, guiding dendrite‐free Li deposition on electrode during cycling. The Fe_2_P_2_O_7_@NC//Li half cells exhibit excellent CE stability across various current densities. Moreover, the symmetric cell with Fe_2_P_2_O_7_@NC‐Li electrodes also demonstrates consistent cycling stability over a period of 1400 h at 1 mA cm^−2^ and 1 mAh cm^−2^ with a small voltage polarization. Particularly, full cells paired by Fe_2_P_2_O_7_@NC‐Li anode and commercial LFP cathodes under low N/P ratios (as low as N/P = 1.0) maintain high capacity retention at high mass loading of cathode, demonstrating that Fe_2_P_2_O_7_@NC is a promising candidate for Li host material in higher‐energy‐density LMBs. This work also validates iron‐based pyrophosphate as a Li host and inspires future designs of advanced Li hosts for LMBs.

## Conflicts of Interest

The authors declare no conflicts of interest.

## Supporting information




**Supporting File**: adma74276‐sup‐0001‐SuppMat.pdf.

## Data Availability

The data that support the findings of this study are available from the corresponding author upon reasonable request.
