# Supplementary material for: Ferrous Pyrophosphate Hollow Nanorods Enable Lithium Metal Batteries Under Low Negative/Positive Capacity Ratios
Source: Adv Mater. 2026 Jul 28;38(52):e74276. doi: 10.1002/adma.74276 (PMC13579164; doi:10.1002/adma.74276)
Supplement: Supplementary file 1 [file ADMA-38-e74276-s001.pdf]

Supporting Information *for*

Ferrous Pyrophosphate Hollow Nanorods Enable Lithium Metal Batteries under Low Negative/Positive Capacity Ratios

*Chen Yu, Jiarui Yang, Deyan Luan, Xiong Wen (David) Lou**

[*] C. Yu, Prof. J. Yang, Prof. D. Y. Luan, Prof. X. W. Lou

Department of Chemistry, City University of Hong Kong, 83 Tat Chee Avenue, Kowloon, 999077, Hong Kong, China   Email: david.lou@cityu.edu.hk

Experimental Section

Chemicals: Iron(III) nitrate nonahydrate ($Fe(NO_3)_3 \cdot 9H_2O$), fumaric acid ($C_4H_4O_4$), phytic acid ($C_6H_{18}O_{24}P_6$) 50 % (w/w) in H_2O, Dopamine hydrochloride ($C_8H_{11}NO_2 \cdot HCl$), Trizma base ($C_4H_{11}NO_3$) were purchased from Sigma-Aldrich without further purification. Li foil (10 mm*0.25 mm), LS-009 ether-based electrolyte (1 M LiTFSI in DME/DOL (v:v = 1:1) with 2 wt% $LiNO_3$ as additive) and $LiFePO_4$ (LFP) powder were purchased from Dodo Chem, Celgard 2325 separator and $Li[Ni_{0.8}Co_{0.1}Mn_{0.1}]O_2$ (NCM811) powder were purchased from Canrd. The ester-based electrolyte (1 M $LiPF_6$ in DMC/EMC/FEC (v:v:v = 1:1:1) with 0.1 M LiDFP) was purchased from Xiamen Shouneng Technology Co., Ltd. Ultrapure water (18.25 mΩ/cm) was used for all experimental process.

Synthesis of MIL-88A Nanorods: the method is based on a reported work.[1] 139.3 mg (1.2 mmol) fumaric acid was introduced into 25 mL deionized water and ultra-sounded for 2 min, then the mixture was moved into a pre-heated oil bath at 70 °C and stirred at 400 rpm for 10 min. Then, 525.2 mg (1.3 mmol) $Fe(NO_3)_3 \cdot 9H_2O$ was introduced to the above mixture and stirred for another 10 min. Next, the mixture was transferred into a Teflon-lined autoclave, heated at 110 °C for 6 h. After the mixture was naturally cooled to room temperature, the solid was collected by centrifugation, followed with washing with deionized water and ethanol and dried in vacuum oven at 60 °C.

Synthesis of PA-Fe hollow nanorods: 100 mg of as-prepared MIL-88A was fully dispersed in 10 mL of deionized water by ultrasonic and stirring, then 15 mL of phytic acid solution (0.2 M) was added into the above solutions with stirring at room temperature, then the solution was moved in a pre-heated oil bath (90 °C) for 3 h with continuous stirring. The product was separated by centrifugation, washed with deionized water and ethanol, and dried in a vacuum oven at 60 °C.

Synthesis of PA-Fe@PDA Hollow nanorods: 60 mg of the as-synthesized PA-Fe nanorods was fully dispersed in 150 mL of 10 mM Tris aqueous buffer, then 90 mg of dopamine hydrochloride was introduced into the above mixture and stirred for 8 h at room temperature. The solid was collected by centrifugation and washed with ethanol for at least 3 times.

Synthesis of $Fe_2P_2O_7$@NC: The as-obtained PA-Fe@PDA was annealed in H_2/Argon (5:95) atmosphere in tube furnace. Specifically, the sample was first annealed at 200 °C for 2 h, then annealed at 400 °C for 3 h, subsequently annealed at 600 °C for 2 h, followed with cooling down to room temperature naturally. The ramping rate was 1 °C min^{-1} throughout the progress.

Synthesis of NC: The NC is obtained by dissolve the as-obtained $Fe_2P_2O_7$@NC in H_2SO_4 solution with heating to remove $Fe_2P_2O_7$.

Materials characterizations: Field-emission scanning electron microscopy (FESEM; Thermo Scientific, Quattro ESEM) was used to examine the morphology of all samples, transmission electron microscopy (TEM, Philips Technai 12 and FEI Tecnai G2 F20) was used to examine the structure of samples. X-ray diffraction (XRD, Bruker D2 Phaser and Bruker D8 Advance) was used to investigate the crystal structure of samples. FESEM and TEM equipment with energy-dispersive X-ray (EDX) spectroscopy was used to examine the elemental composition of samples. The scanning electron microscopy (STEM) and elemental mapping images of TEM were collected by FEI Tecnai G2 F20. The electronic structure and valence state of samples were analyzed by X-ray photoelectron spectroscopy (XPS, Thermo Scientific K-Alpha). Raman spectrum was examined on a Horiba LabRAM HR Evolution Raman microscope. Time-of-flight secondary ion mass spectrometer (TOF-SIMS) was used to investigate the chemical composition distribution with sputtering times on a TOF-SIMS IONTOF M6 instrument (IONTOF GmbH). The in-situ Li deposition process is observed

through optical microscopy (Leica DVM6). For the FESEM, TEM, TOF-SIMS and XPS investigation, the Li-plated or post-cycling electrodes were carefully rinsed by DME and dried in glove box in advance, followed with transferring in inert atmosphere.

Electrochemical measurements: The electrochemical measurements were carried by assembling CR2032 coin cells and tested on a NEWARE battery tester (CT-4008-5 V 10 mA-164). Celgard 2325 was employed as separator. LS-009 ether-based electrolyte (1 M LiTFSI in DME/DOL (v:v = 1:1) with 2 wt% $LiNO_3$ as additive) was used as electrolyte. For preparing $Fe_2P_2O_7$@NC-Cu electrode, $Fe_2P_2O_7$@NC, Super P-Li and polyvinylidene fluoride were mixed in a weight ratio of 8:1:1, slurry by using *N*-methyl-2-pyrrolidone as solvent was cast onto a Cu foil and dried at 80 °C in a vacuum oven. Half cells assembled by $Fe_2P_2O_7$@NC-Cu or b-Cu (working electrode), and Li foil (counter electrode) was used to investigate CE of Li plating/stripping process. For symmetric cells cycling test, $Fe_2P_2O_7$@NC-Cu with Li pre-plated ($Fe_2P_2O_7$@NC-Li) was employed as electrodes. For full cell tests, commercial LFP powder, polyvinylidene fluoride and Super P-Li were mixed in a weight ratio of 8:1:1, slurry by using *N*-methyl-2-pyrrolidone as solvent was casted onto an Al foil and dried at 150 °C in vacuum oven. The mass loading of LFP cathode is ~7.46 mg cm^{-2}. The prepared LFP cathodes were paired with $Fe_2P_2O_7$@NC-Li, NC-Li and Cu-Li anodes with different N/P capacity ratios to assemble full cells. LFP full cells for rate capability testing were pre-activated with 2 cycles at 0.2 C. Besides, we replaced the LFP powder with a NCM811 powder and followed the same procedure to prepare the NCM811 cathode and assembled the full cell. The mass loading of NCM811 cathode is ~3.4 mg cm^{-2}. Except for the NCM full cells that used ester-based electrolyte, all other cells used ether-based electrolyte. The NCM811 full cell was activated by two preliminary cycles at

0.1 C before formal cycling. All the cells were assembled in a glove box with H_2O/O_2 content lower than 0.01 ppm, and the electrolyte used in each cell was controlled to be about 80 μL.

In-situ FT-IR testing: The in-situ FT-IR testing was carried out on a Bruker V80 instrument with a Mercury-Cadmium-Telluride (MCT) detector. The angle of incident is set as 60°. Before the in-situ FT-IR testing, in-situ half cell was assembled by $Fe_2P_2O_7$@NC-Cu electrode (working electrode) and Li foil (counter electrode) in a glove box (H_2O/O_2 content lower than 0.01 ppm), followed with plating 1 mAh cm^{-2} Li on $Fe_2P_2O_7$@NC-Cu to obtain $Fe_2P_2O_7$@NC-Li//Li half cell. Next, plated Li on $Fe_2P_2O_7$@NC-Li electrode in the above cell was stripped potentiostatically at 0.1 V for 800 sec by using a CHI 760E electrochemistry workstation, and the in-situ FT-IR spectrum data of this process were recorded.[2]

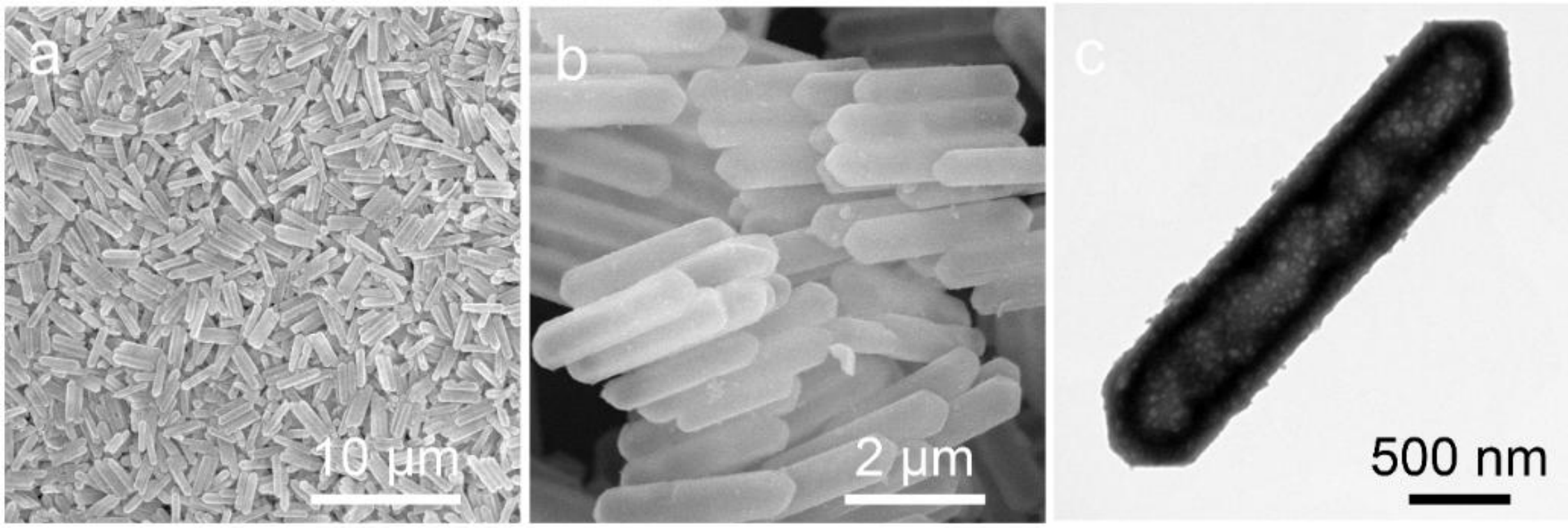

Figure S1. (a, b) FESEM and (c) TEM images of PA-Fe hollow nanorods.

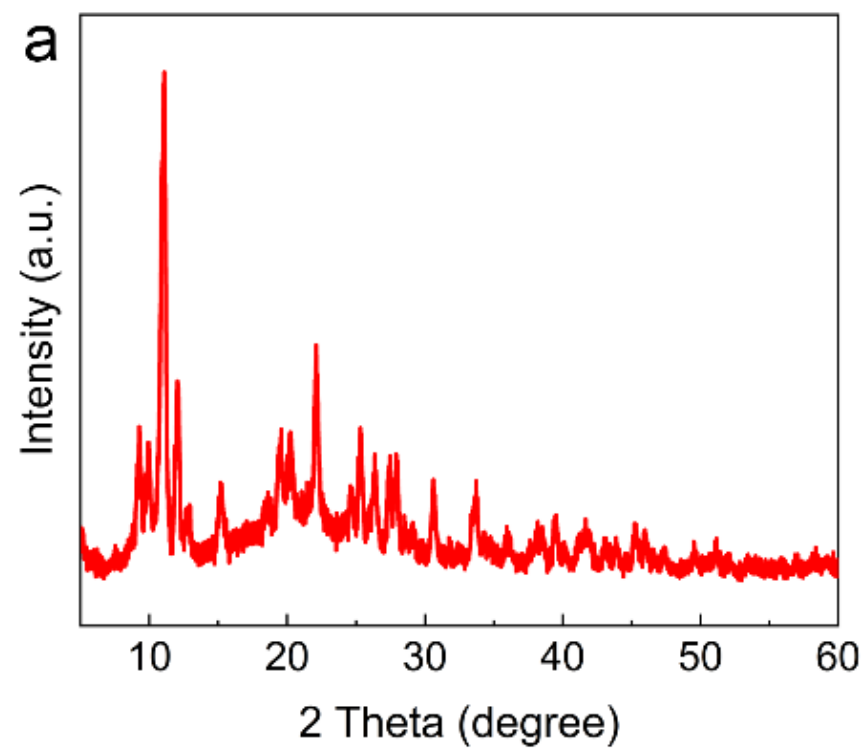

Figure S2. (a) XRD pattern and (b) EDX spectrum of MIL-88A (The signal of Si is from the substrate).

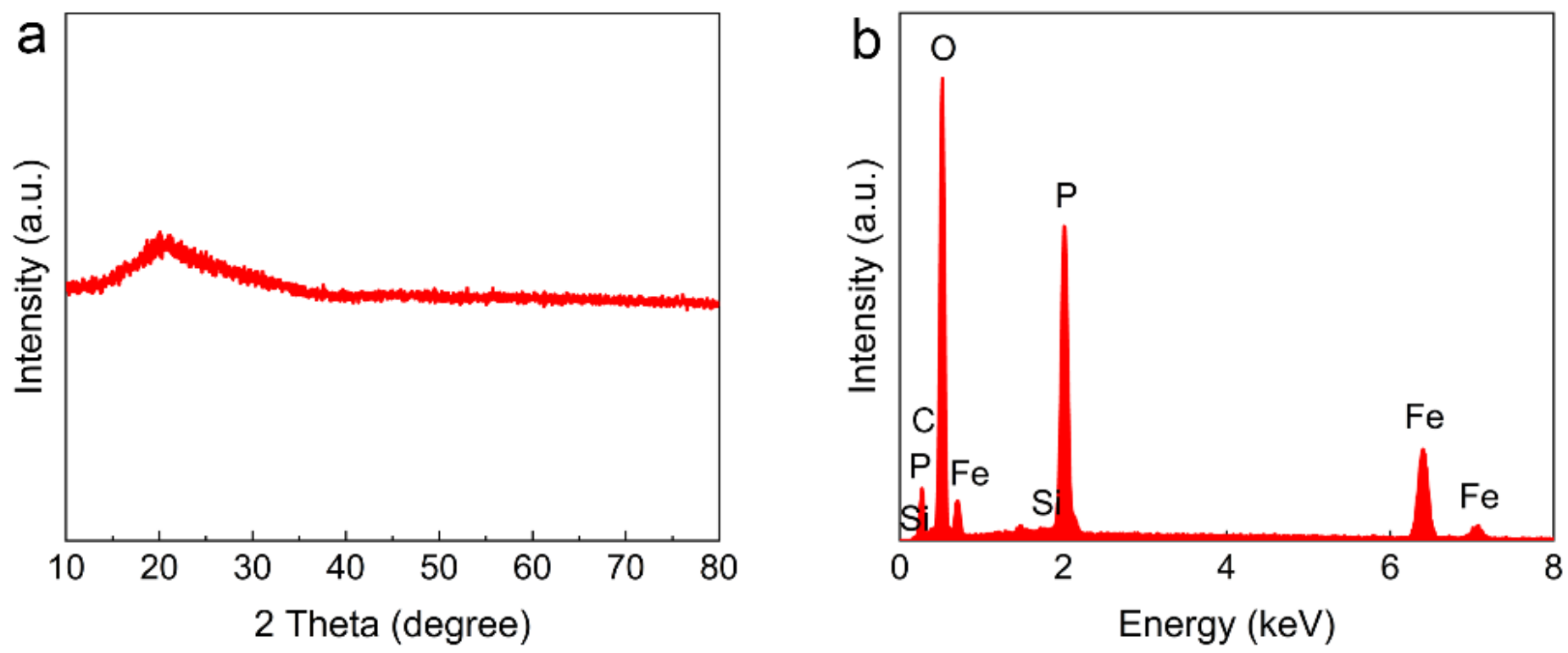

Figure S3. (a) XRD pattern and (b) EDX spectrum of PA-Fe hollow nanorods (The signal of Si is from the substrate).

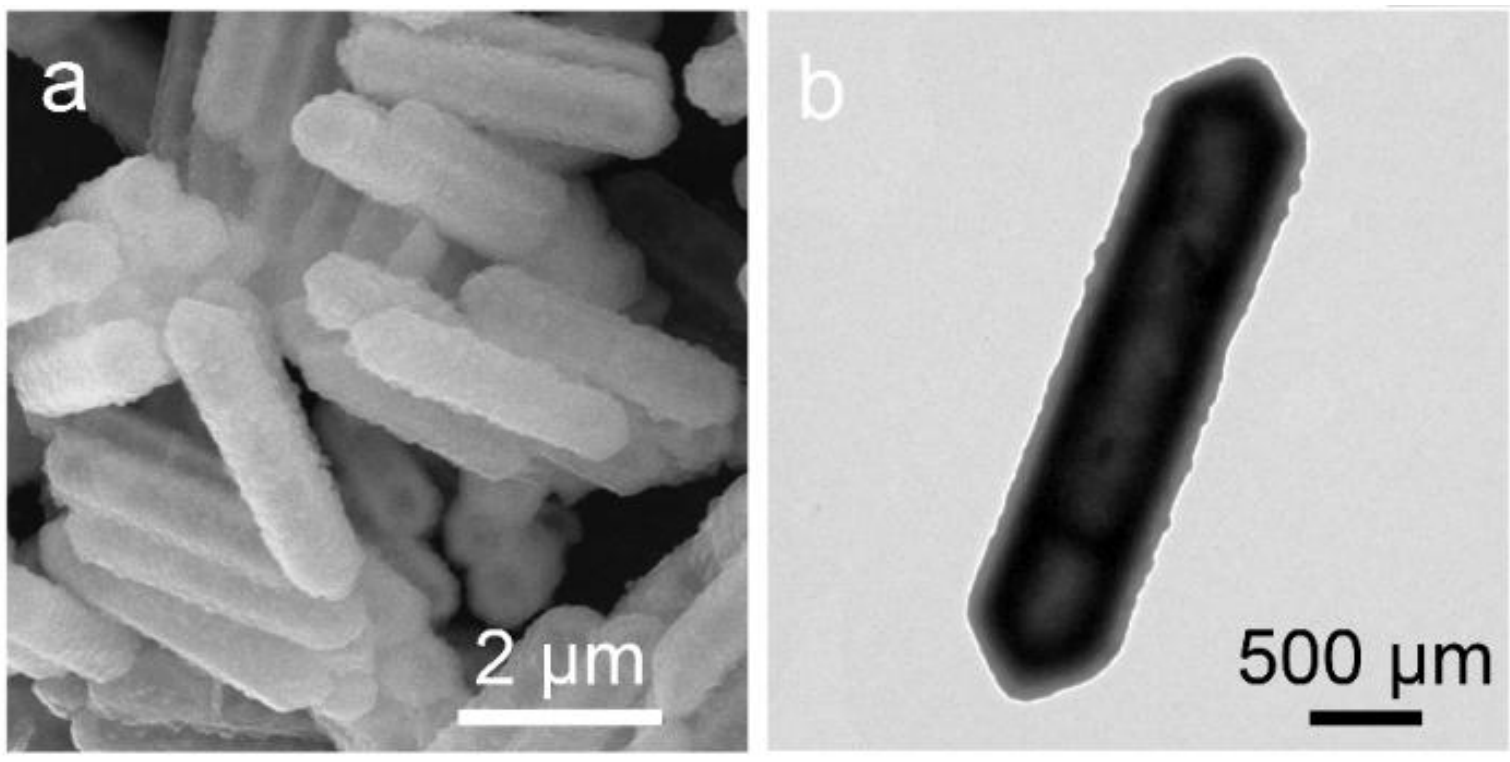

Figure S4. (a) FESEM and (b) TEM images of PA-Fe@PDA hollow nanorods.

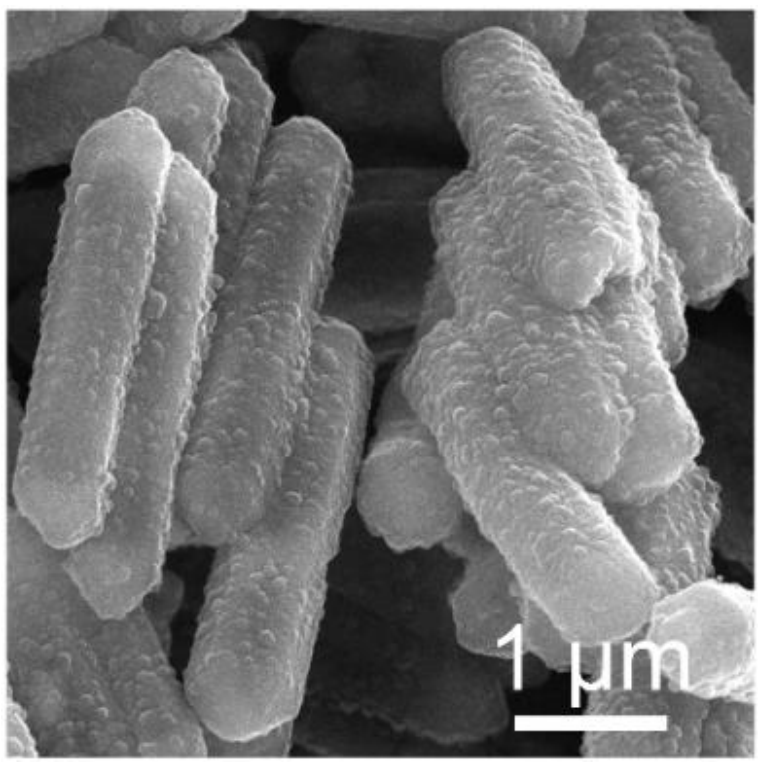

Figure S5. FESEM image of multiple $Fe_2P_2O_7$@NC with lower magnification.

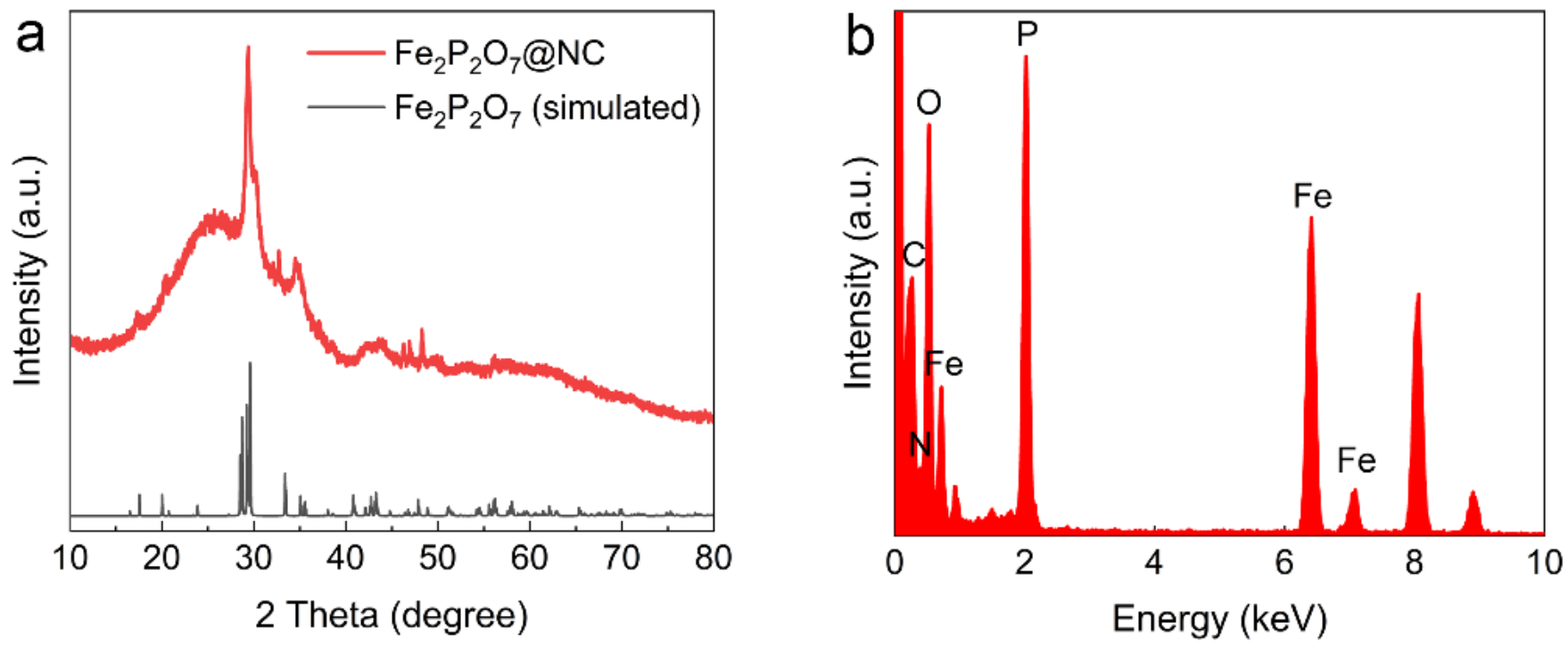

Figure S6. (a) XRD pattern and (b) EDX spectrum of $Fe_2P_2O_7$@NC.

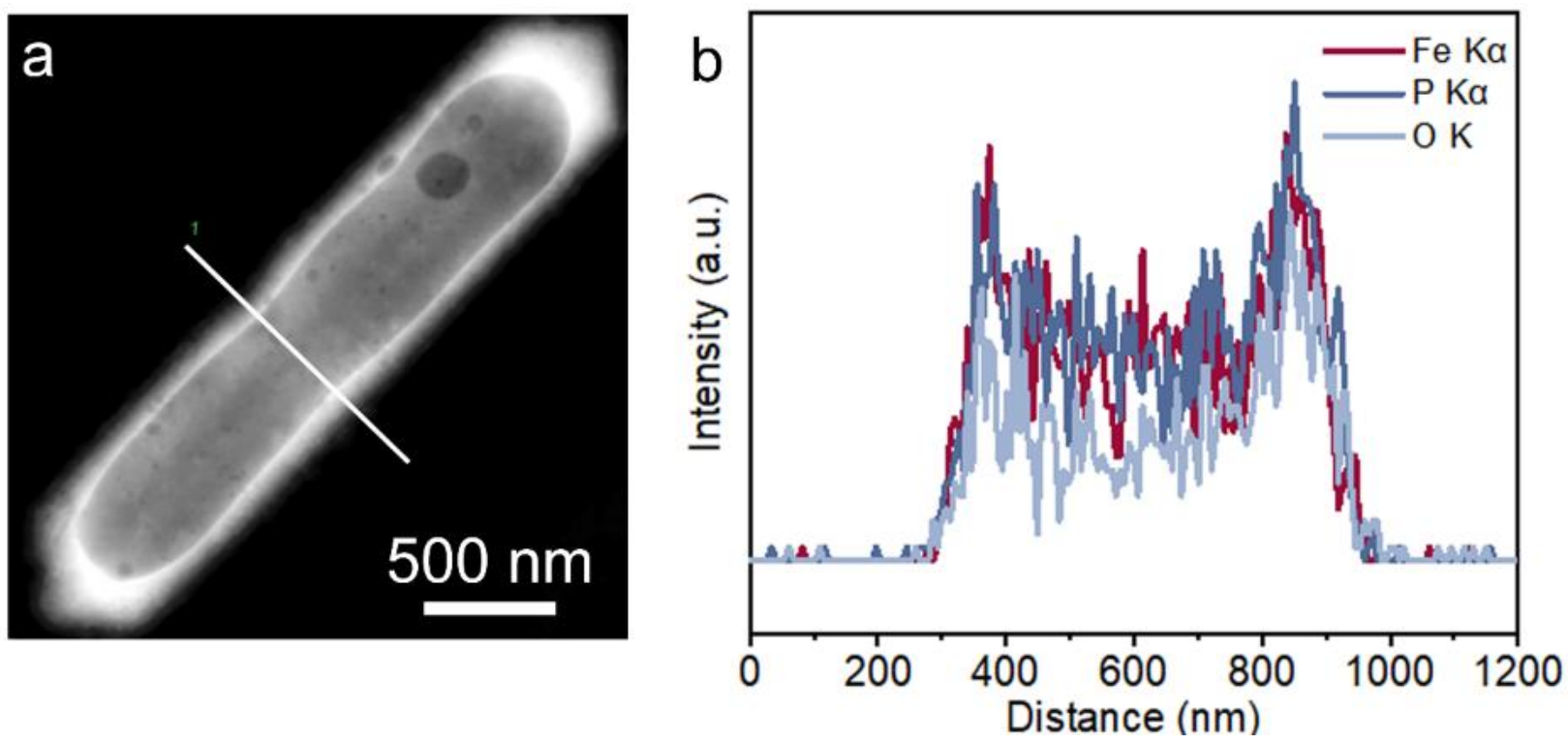

Figure S7. (a) HAADF-STEM image of $Fe_2P_2O_7$@NC. (b) Fe, P, and O element distribution of $Fe_2P_2O_7$@NC by EDX line scanning.

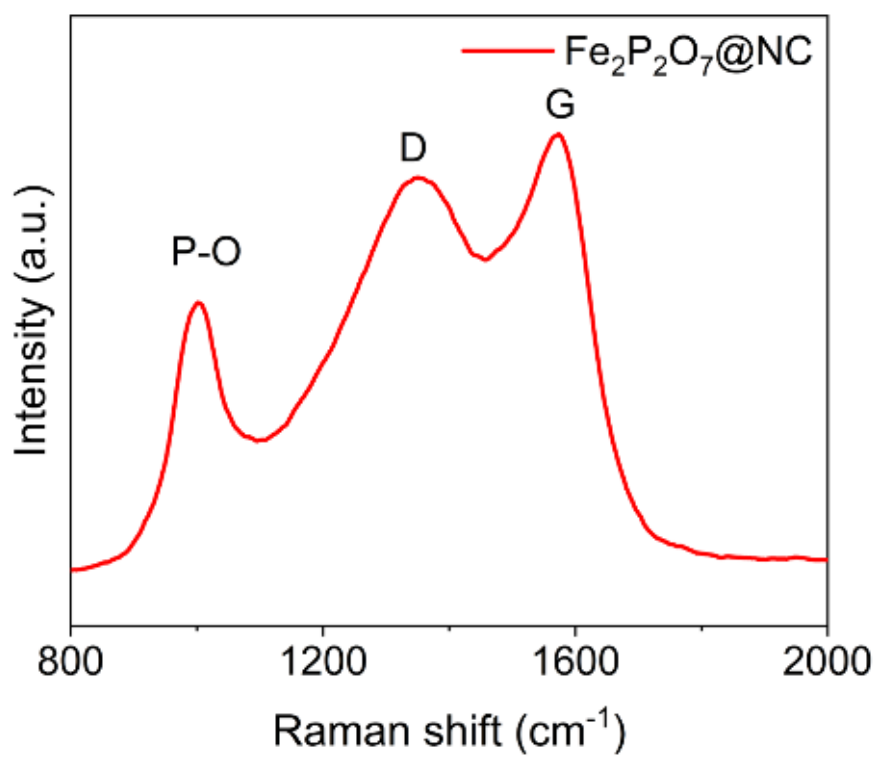

Figure S8. Raman spectrum of $Fe_2P_2O_7$@NC.

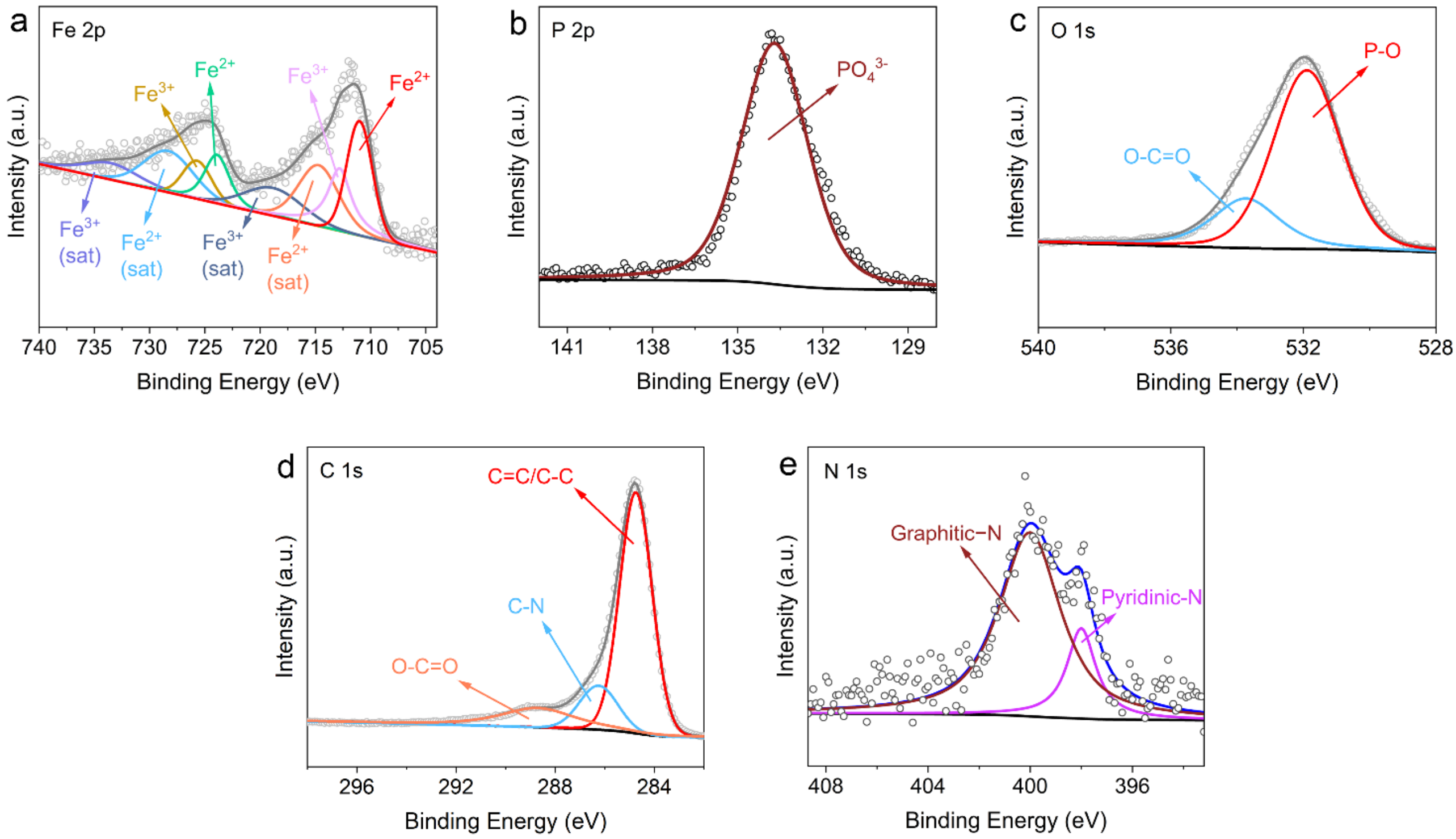

Figure S9. XPS measurement. (a) Fe 2p, (b) P 2p, (c) O 1s, (d) C 1s, and (e) N 1s XPS spectra of $Fe_2P_2O_7$@NC.

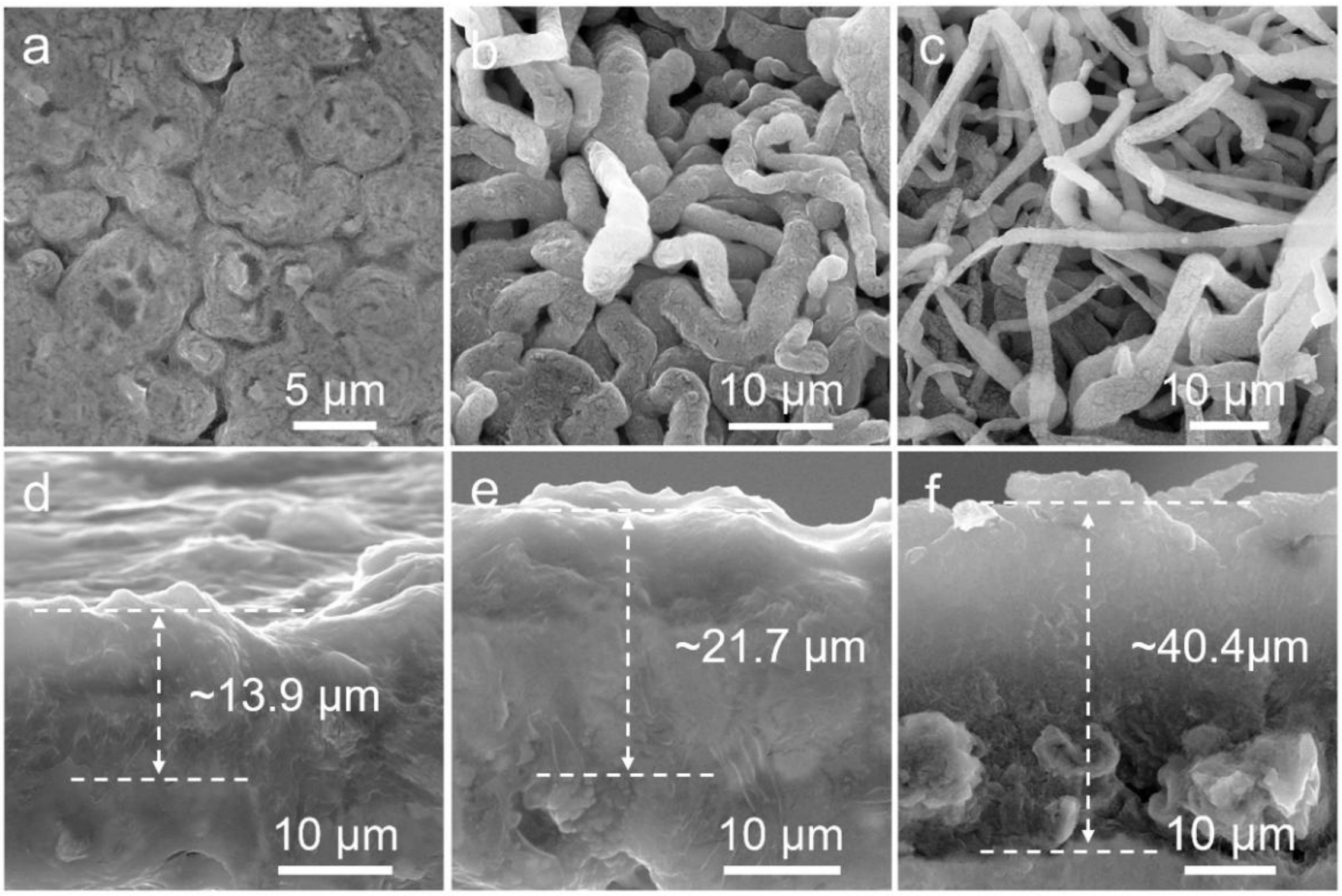

Figure S10. Li deposition behavior on the b-Cu. (a-c) Top and (d-e) cross-sectional FESEM images of Li deposited with a capacity of (a, d) 1 mAh cm^{-2}, (b, e) 3 mAh cm^{-2}, (e, f) 5 mAh cm^{-2}.

Note: these results are obtained by subtracting the thickness of the Cu foil (10 μm) from the total thickness of the electrode cross-section).

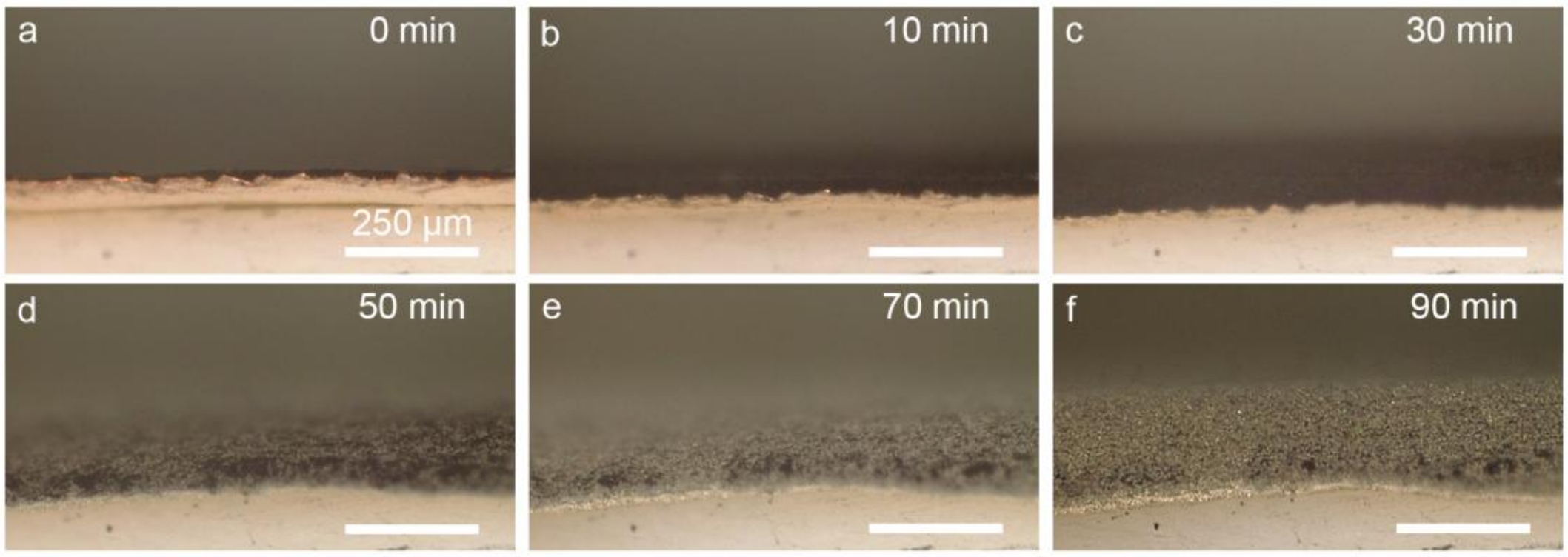

Figure S11. In-situ optical microscopy images of Li deposition on a $Fe_2P_2O_7$@NC-Cu electrode at 3 mA cm-2 with increasing discharging time. The scale bars in (b-f) are 250 μm.

Note: During the testing, the side of $Fe_2P_2O_7$@NC-Cu electrode which near the observation window exhibited a slight downward tilt. Nevertheless, the evenness of Li deposition and the thickness variation of the electrode are still clearly discernible.

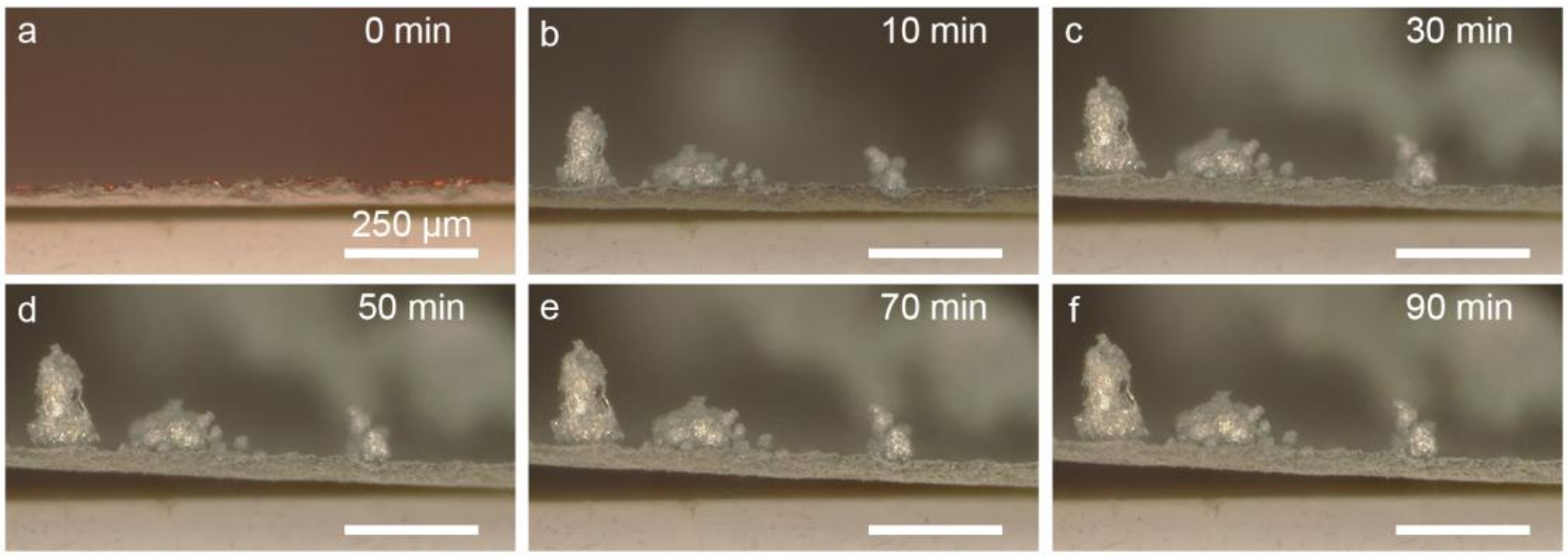

Figure S12. In-situ optical microscopy images of Li deposition on a b-Cu electrode at 3 mA cm^{-2} with increasing discharging time. The scale bars in (b-f) are 250 µm.

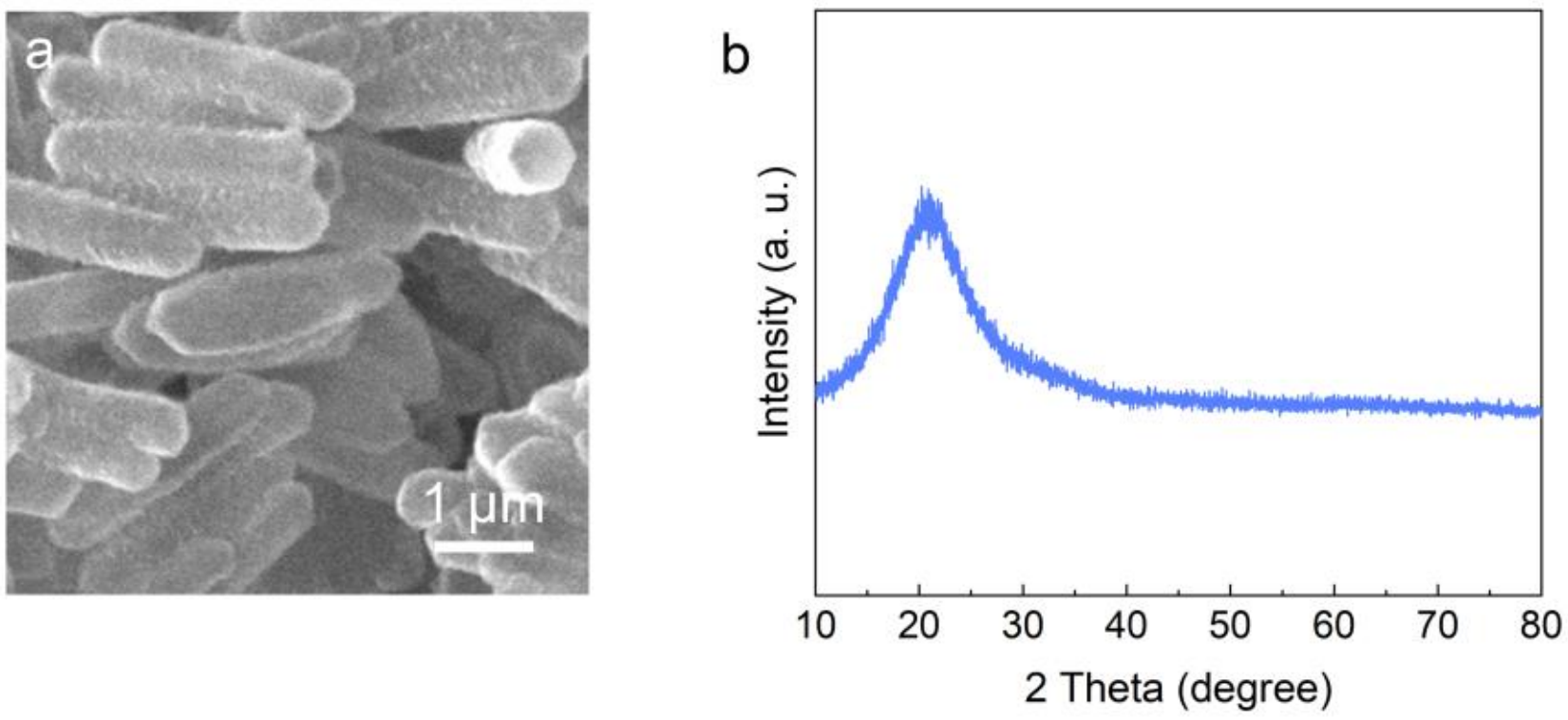

Figure S13. FESEM image and XRD pattern of NC.

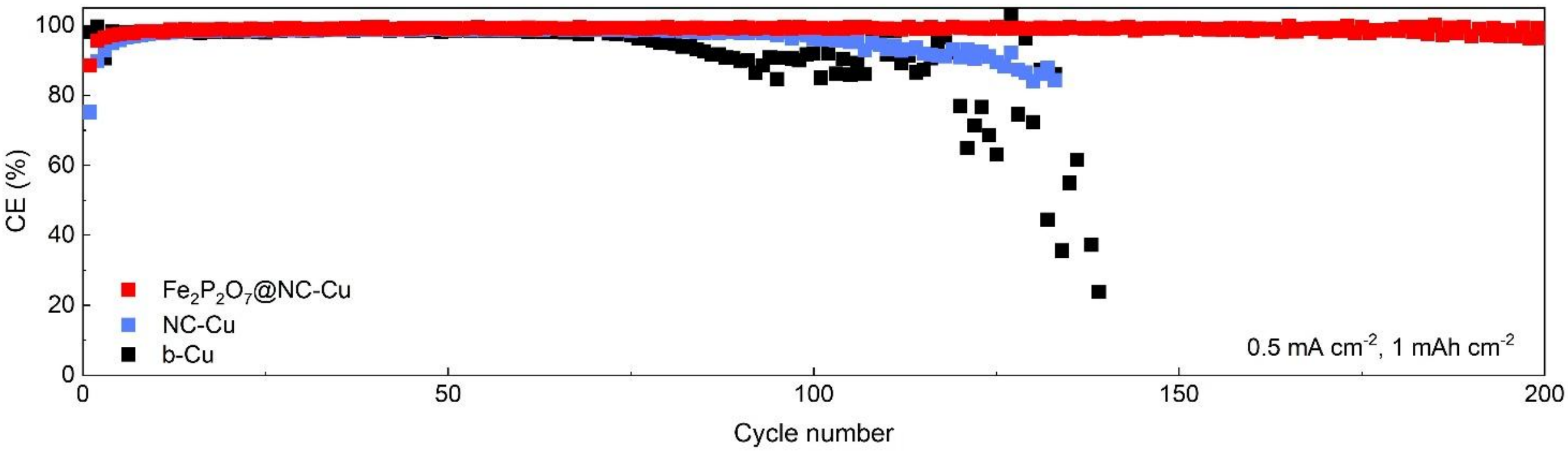

Figure S14. CE comparison of different electrodes at 0.5 mA cm^{-2} and 1 mAh cm^{-2}.

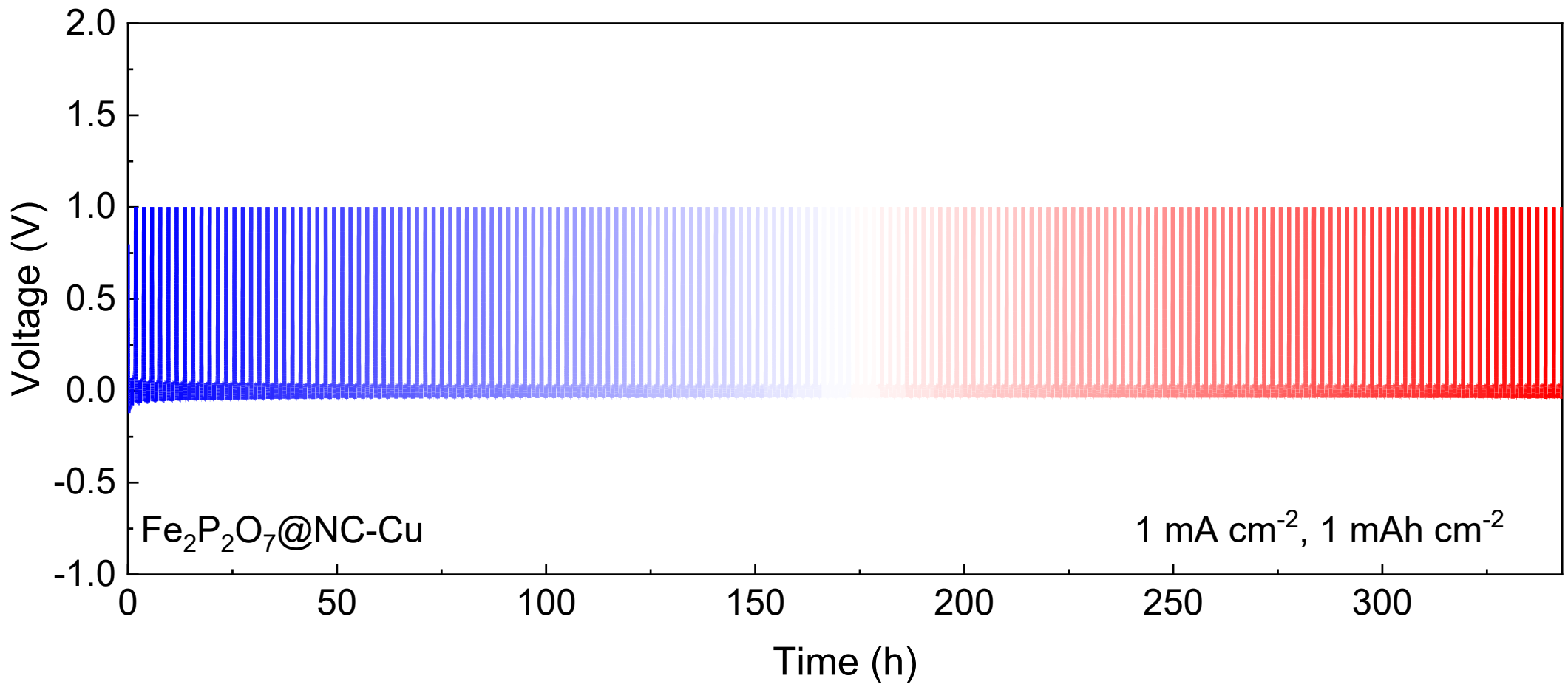

Figure S15. Voltage-time curve of $Fe_2P_2O_7$@NC-Cu//Li cell cycle at 1 mA cm^{-2} and 1 mAh cm^{-2}.

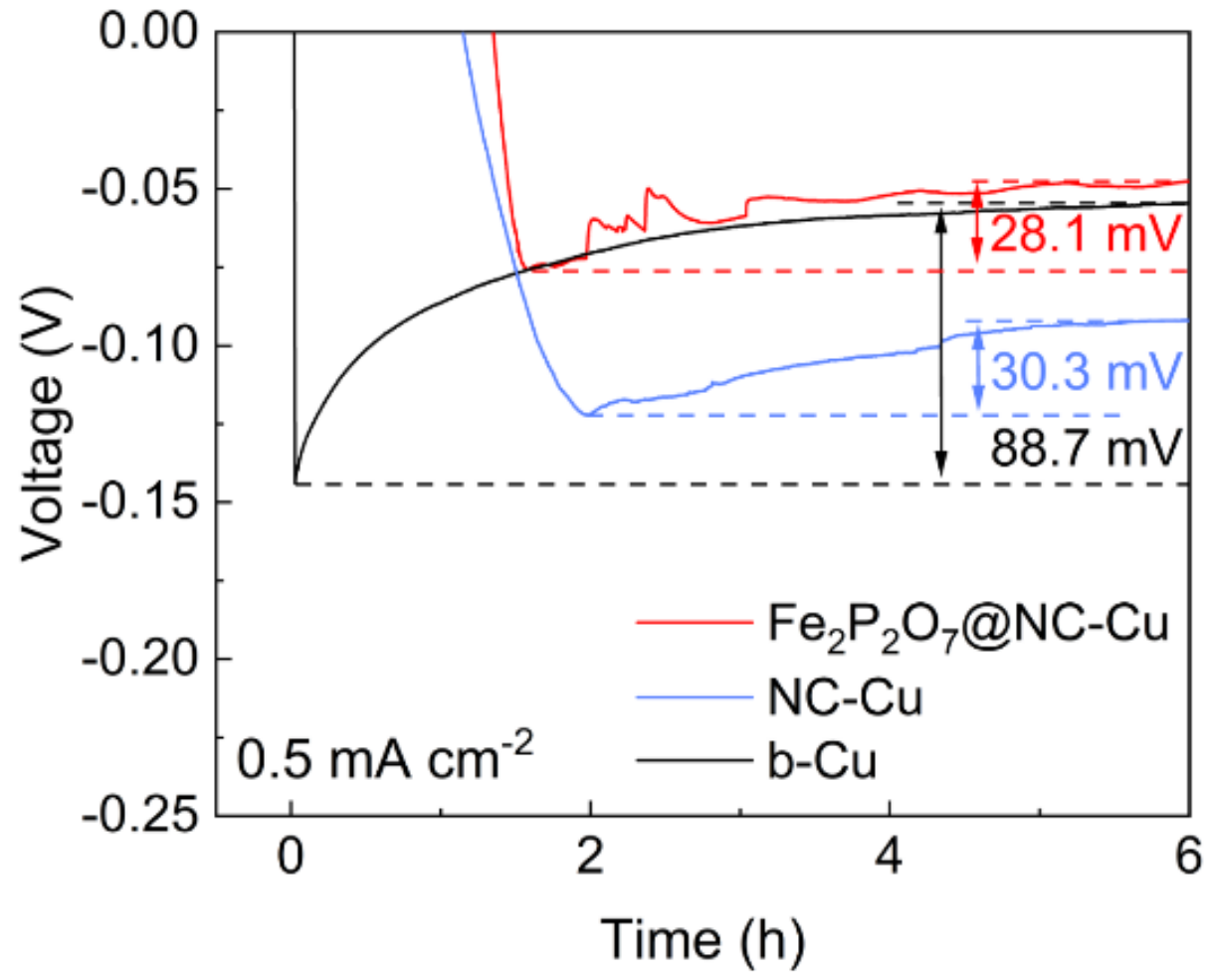

Figure S16. Voltage-time profiles of Li deposition at 0.5 mA cm^{-2} on different electrodes.

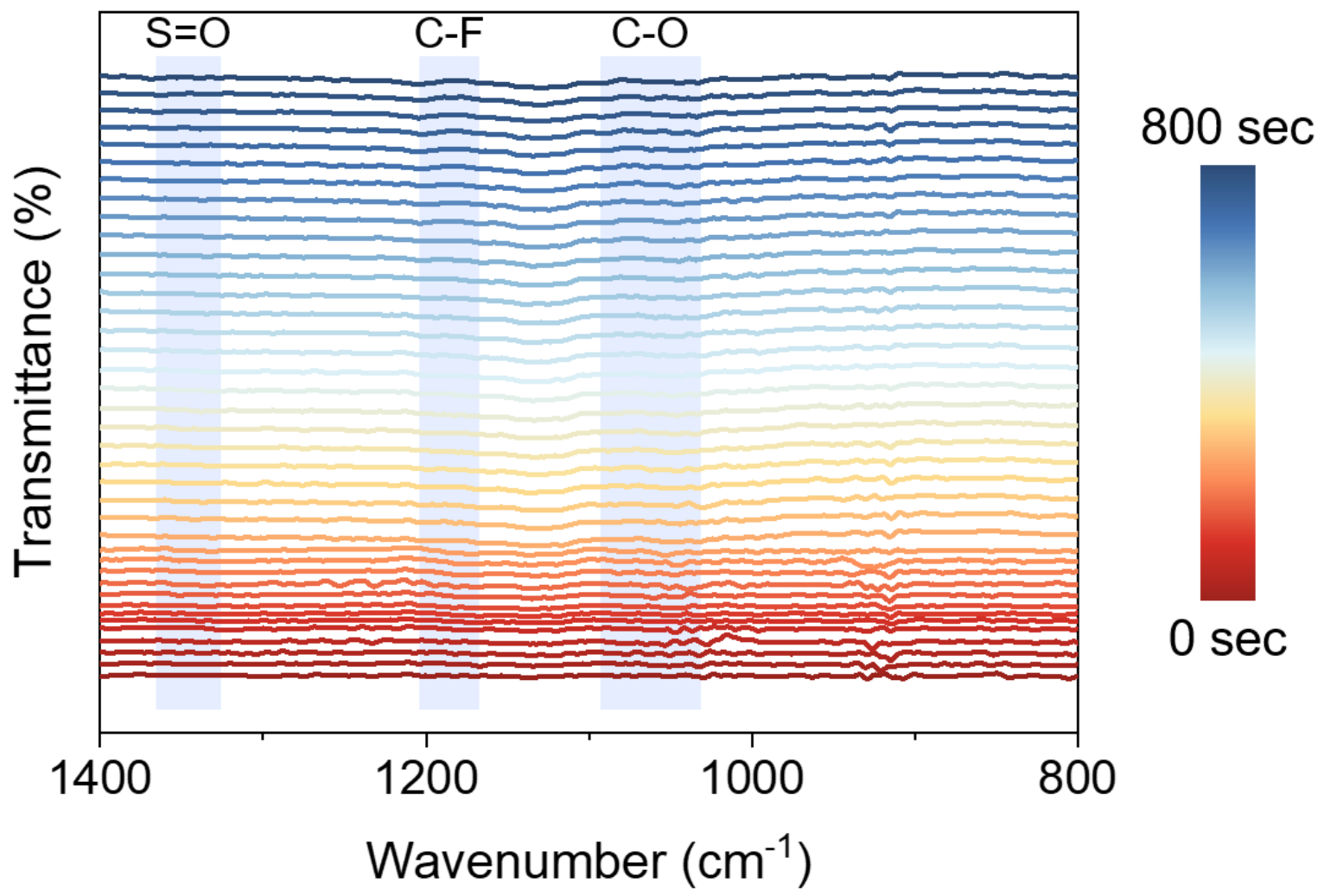

Figure S17. In-situ FT-IR spectra of $Fe_2P_2O_7$@NC-Cu//Li cell during Li stripping process.[2]

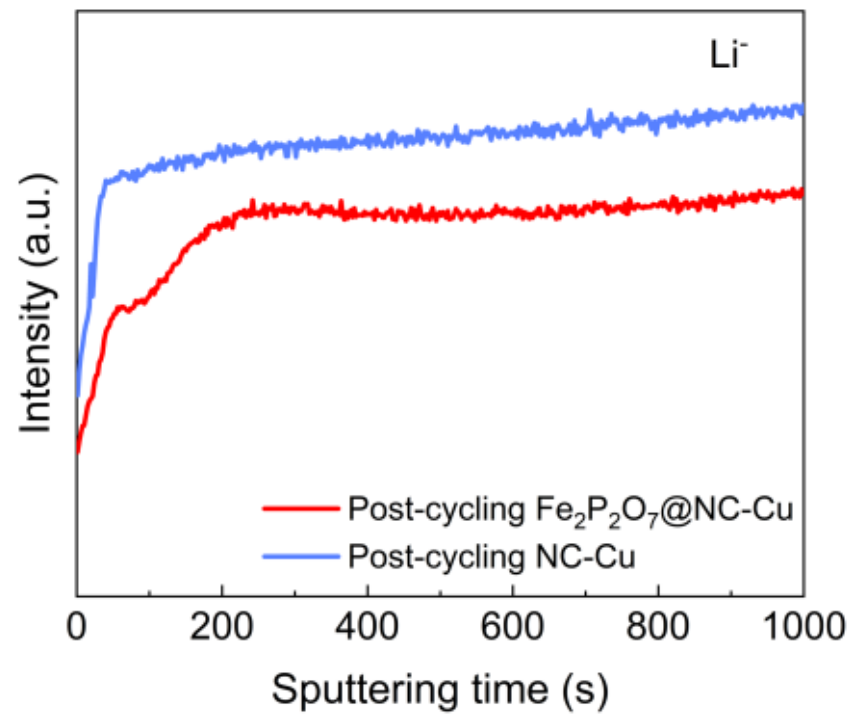

Figure S18. Depth-resolved intensity profiles of Li^- of post-cycling $Fe_2P_2O_7$@NC-Cu and NC-Cu electrodes.

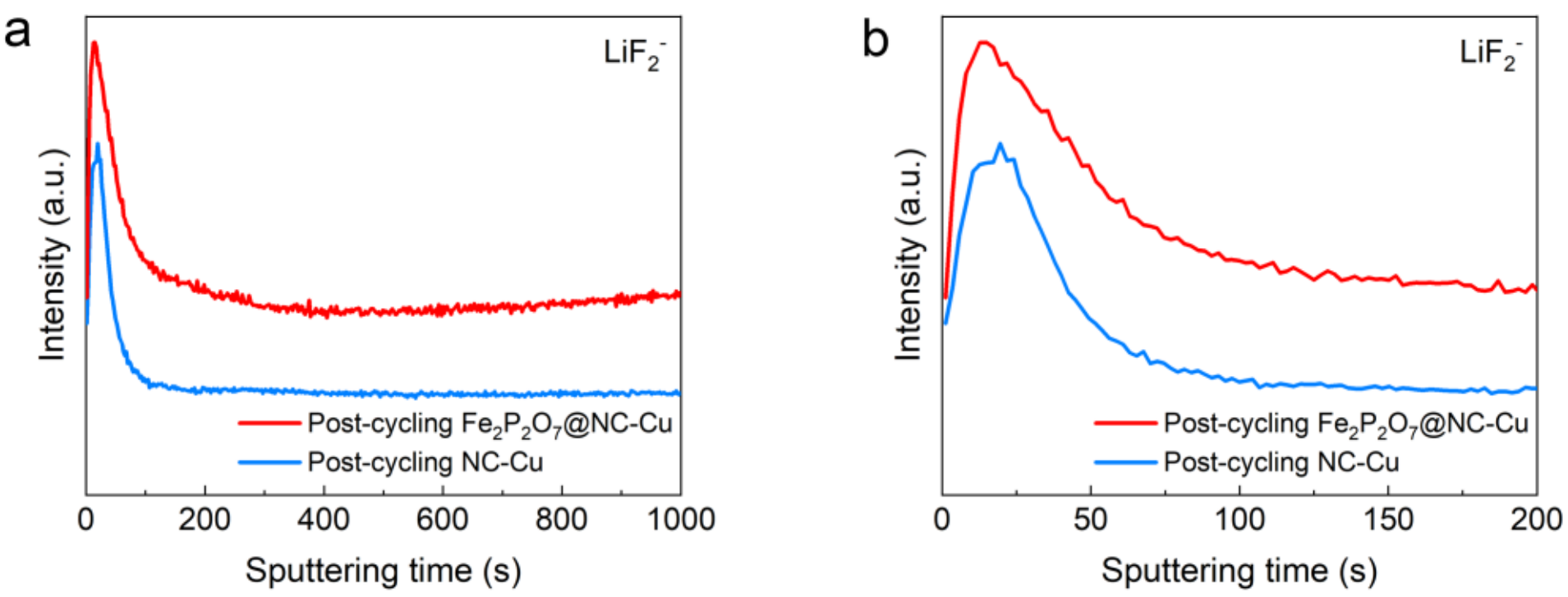

Figure S19. (a) Depth-resolved intensity profiles of LiF_2^- of post-cycling $Fe_2P_2O_7$@NC-Cu and NC-Cu electrodes and (b) corresponding local magnified image.

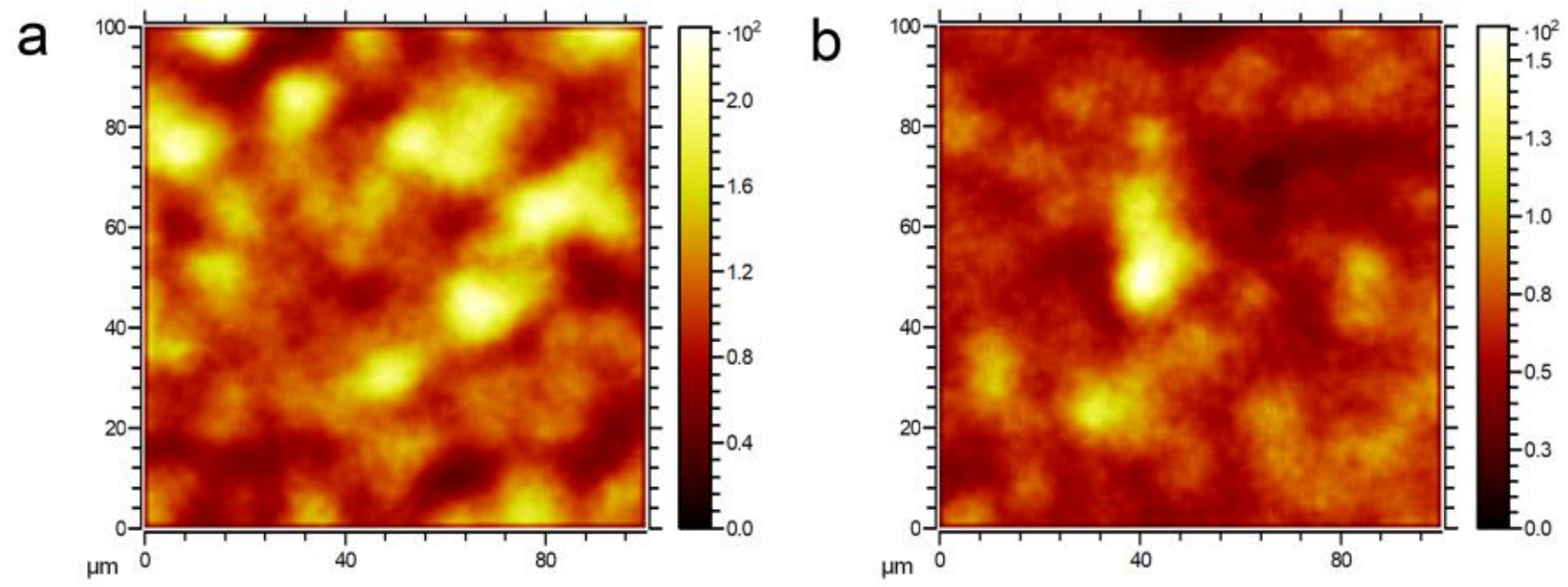

Figure S20. TOF-SIMS negative ion mapping of LiF_2^- of the post-cycling (a) $Fe_2P_2O_7$@NC -Cu and (b) NC-Cu electrodes.

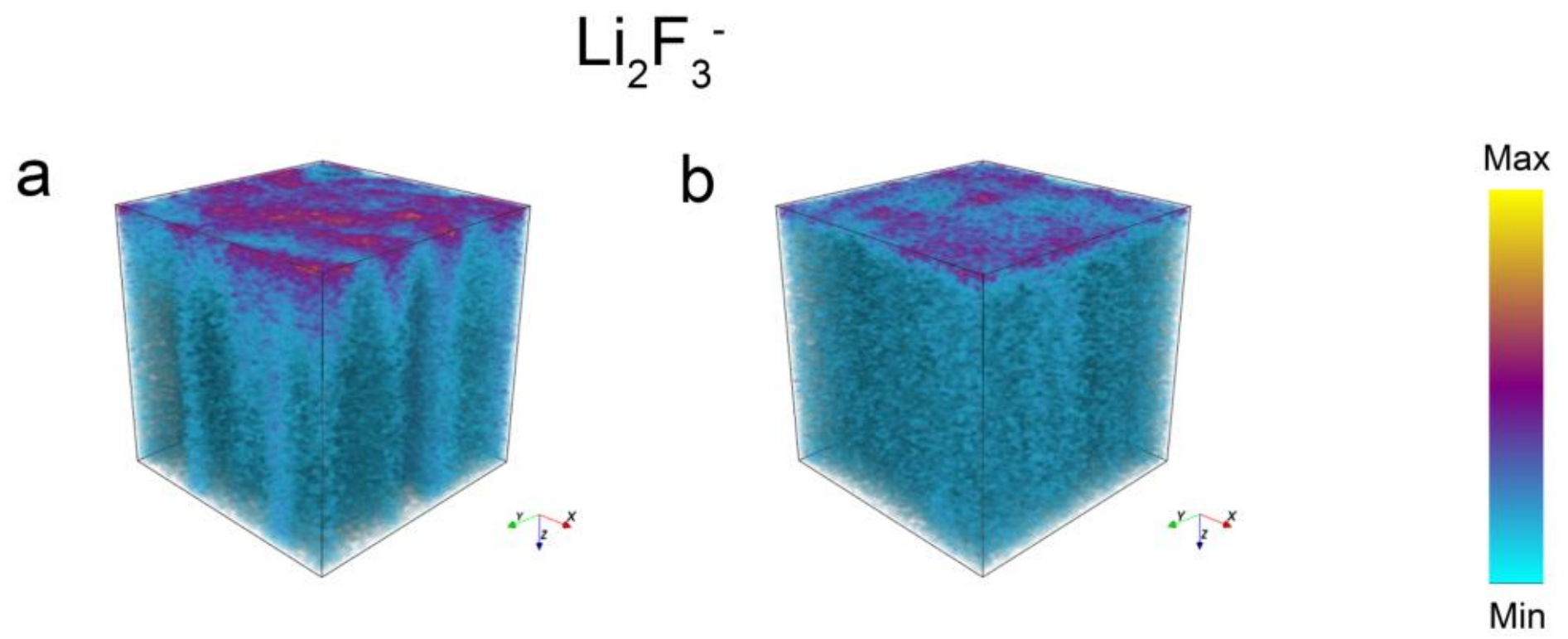

Figure S21. TOF-SIMS characterization of $Li_2F_3^-$ of (a) $Fe_2P_2O_7$@NC@NC-Cu and (b) NC-Cu electrodes after cycled for 100 cycles.

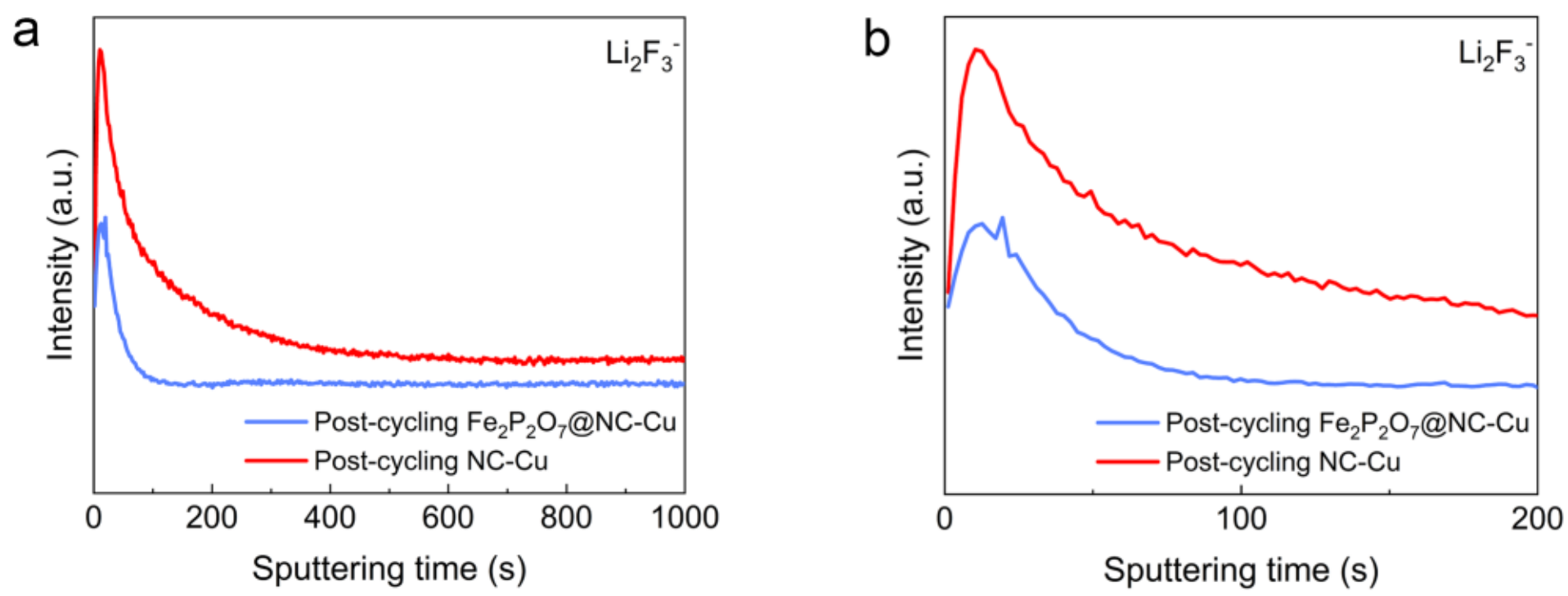

Figure S22. (a) Depth-resolved intensity profiles of $Li_2F_3^-$ of post-cycling $Fe_2P_2O_7$@NC@NC-Cu electrode and (b) corresponding local magnified image.

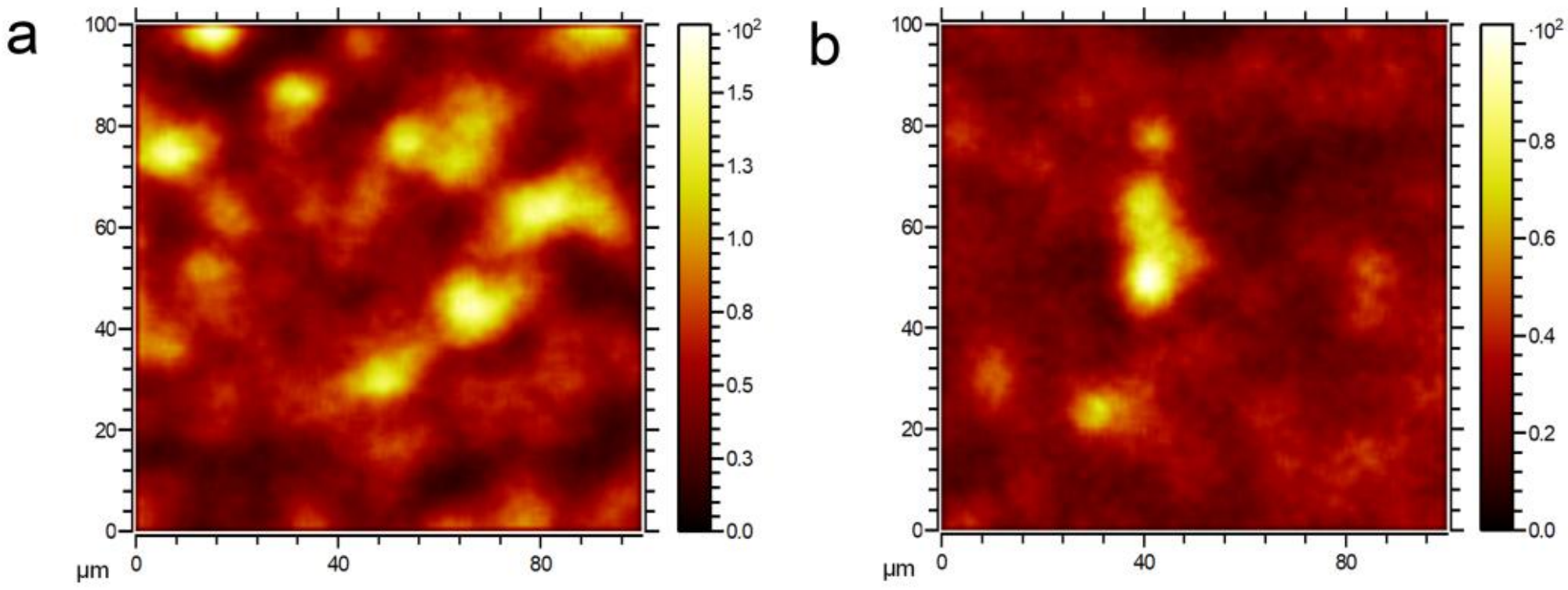

Figure S23. TOF-SIMS negative ion mapping of $Li_2F_3^-$ of the post-cycling (a) $Fe_2P_2O_7$@NC@NC-Cu and (b) NC-Cu electrodes.

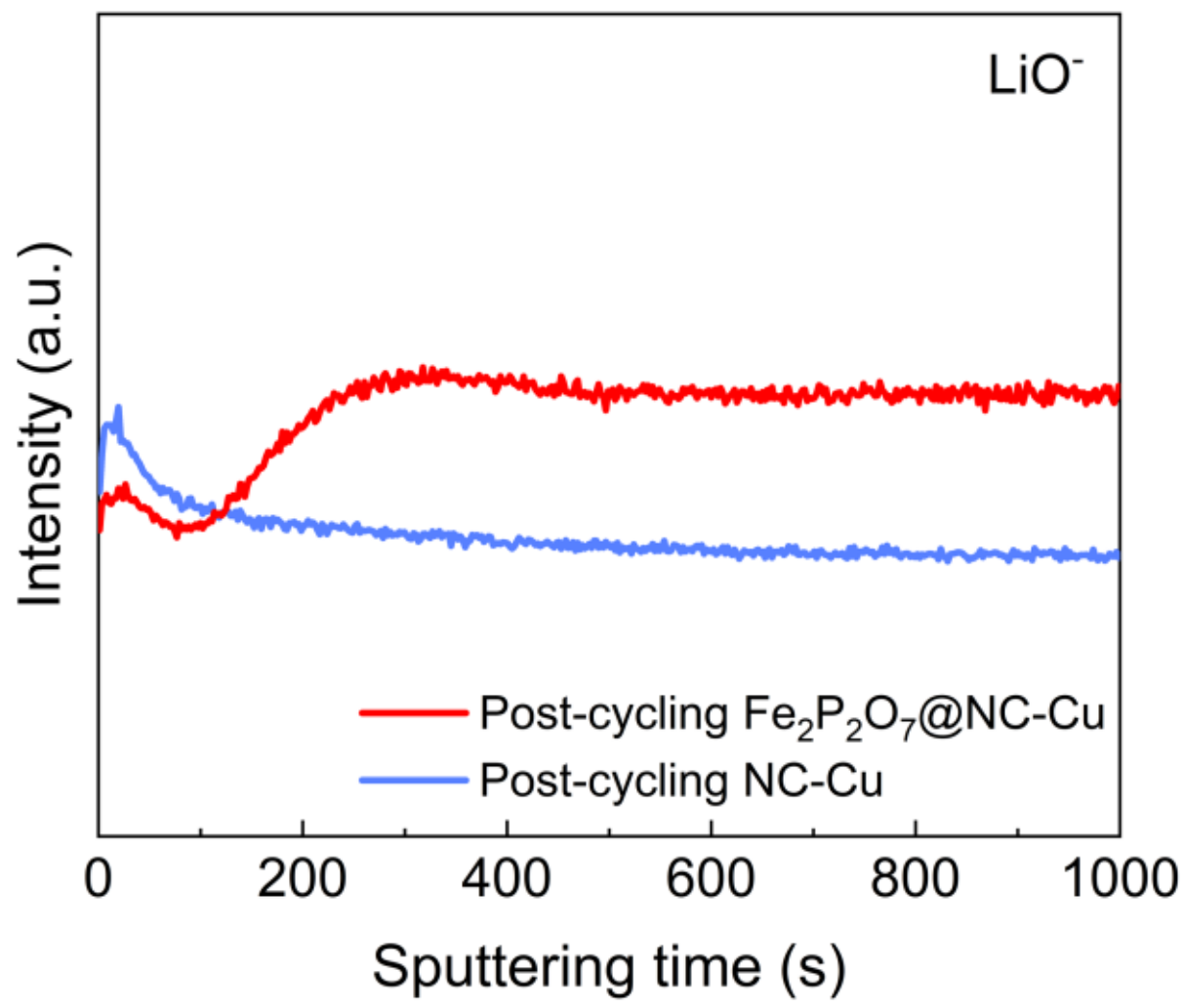

Figure S24. Depth-resolved intensity profiles of LiO^- of post-cycling $Fe_2P_2O_7$@NC-Cu and NC-Cu electrodes.

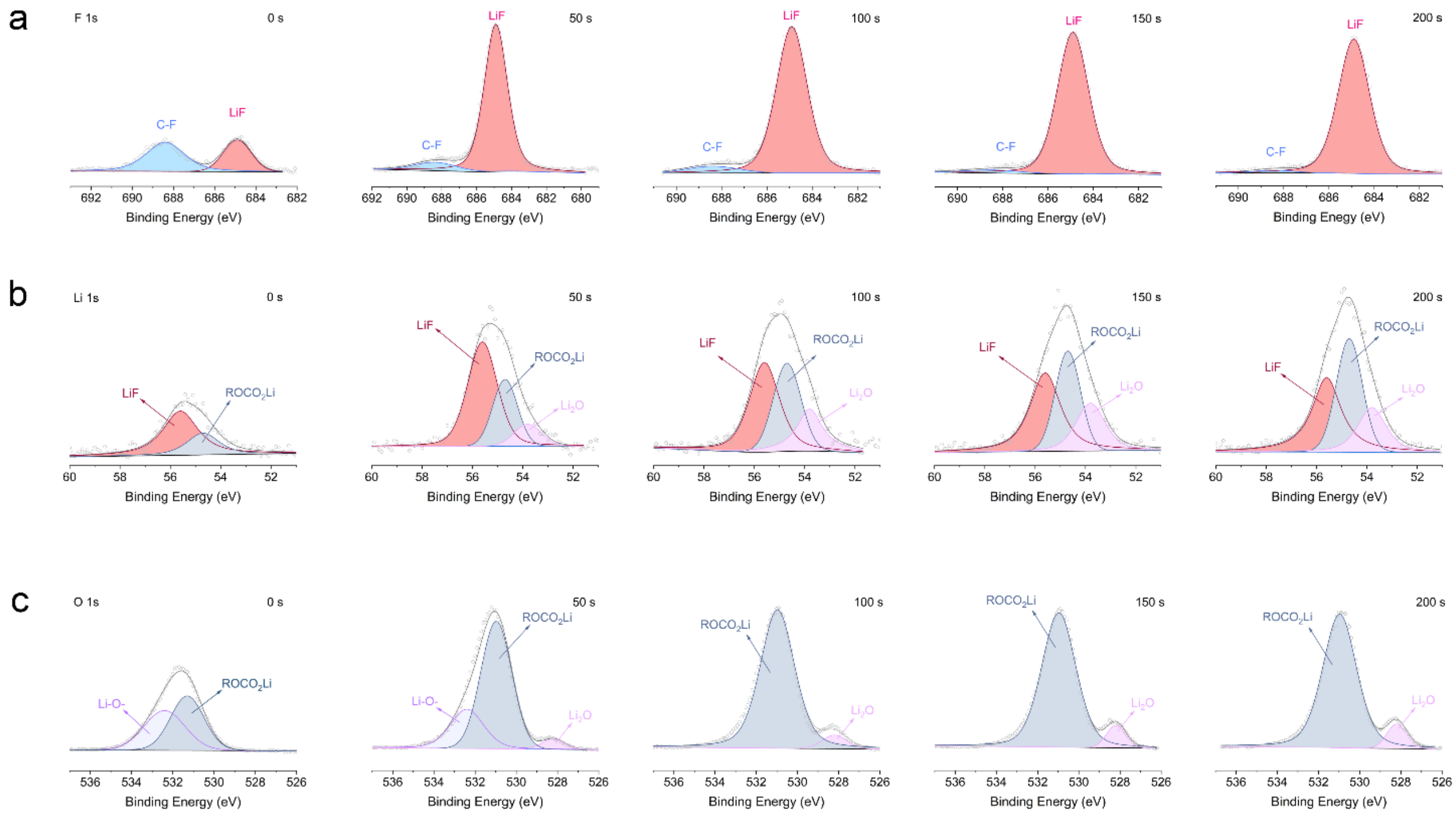

Figure S25. In-depth XPS analysis of post-cycling $Fe_2P_2O_7$@NC-Cu electrode. (a) F 1s (b) Li 1s (c) O 1s spectra.

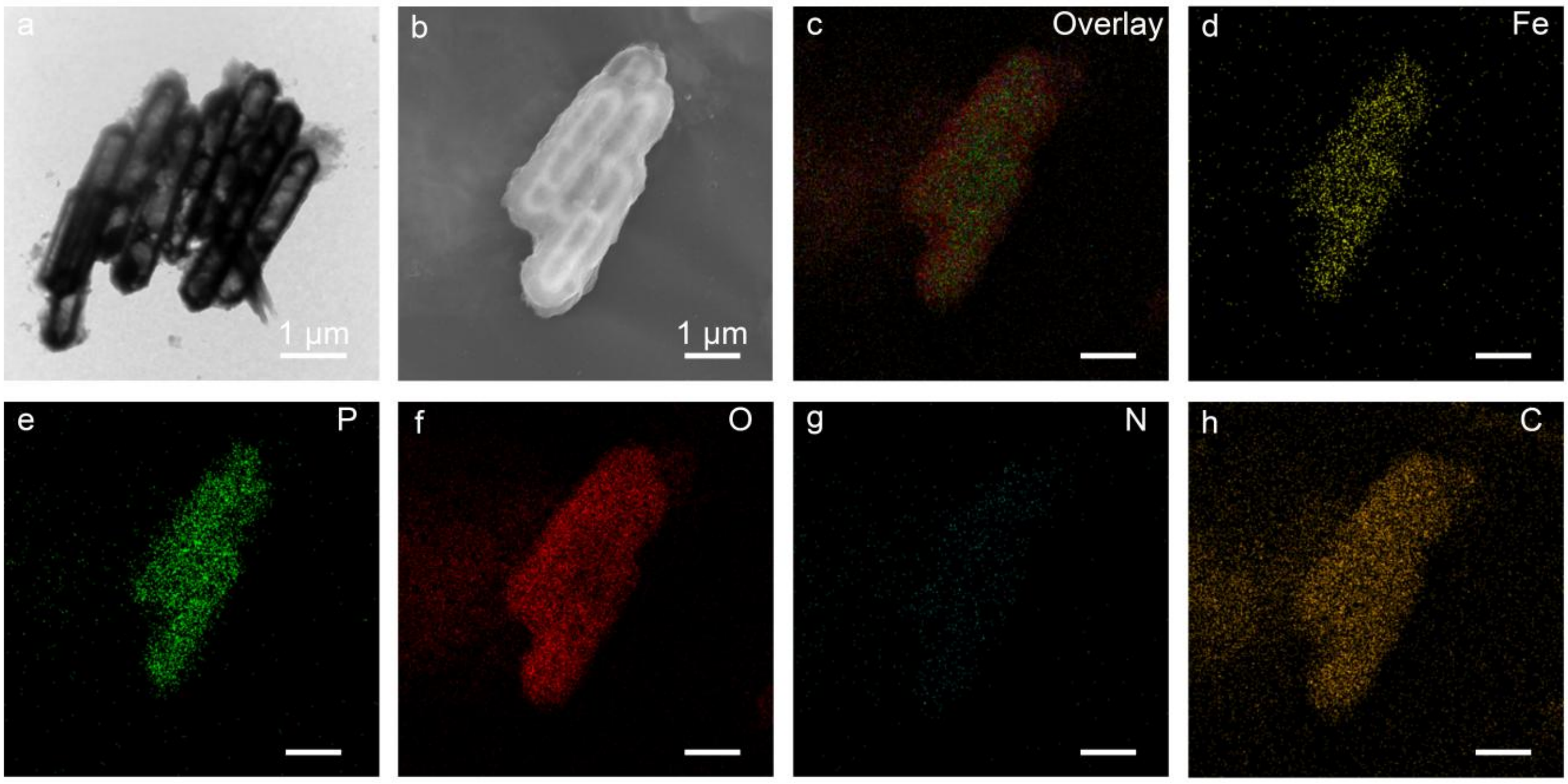

Figure S26. Structure and elemental distribution investigation of $Fe_2P_2O_7$@NC nanorods cycled at 2 mA cm^{-2} and 1 mAh cm^{-2} for 100 cycles in a half cell. (a) TEM image of the post-cycling $Fe_2P_2O_7$@NC nanorods. FESEM image (b) and corresponding elemental mapping images (c-h) of the post-cycling $Fe_2P_2O_7$@NC nanorods. Scale bars in (c-h): 1 μm.

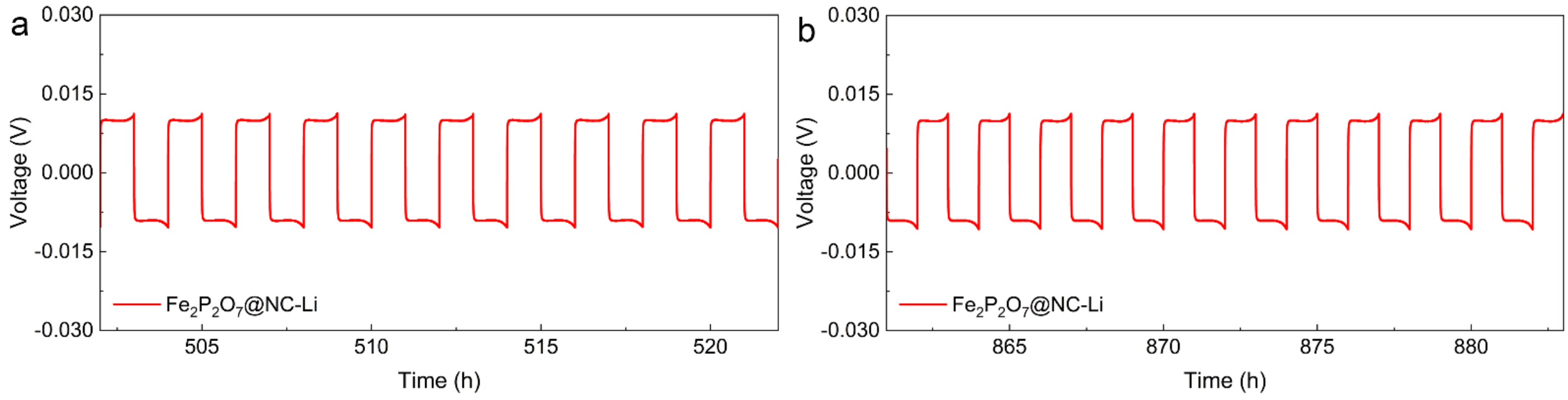

Figure S27. Enlarged voltage profiles of $Fe_2P_2O_7$@NC-Li tested at 1 mA cm^{-2} and 1 mAh cm^{-2}.

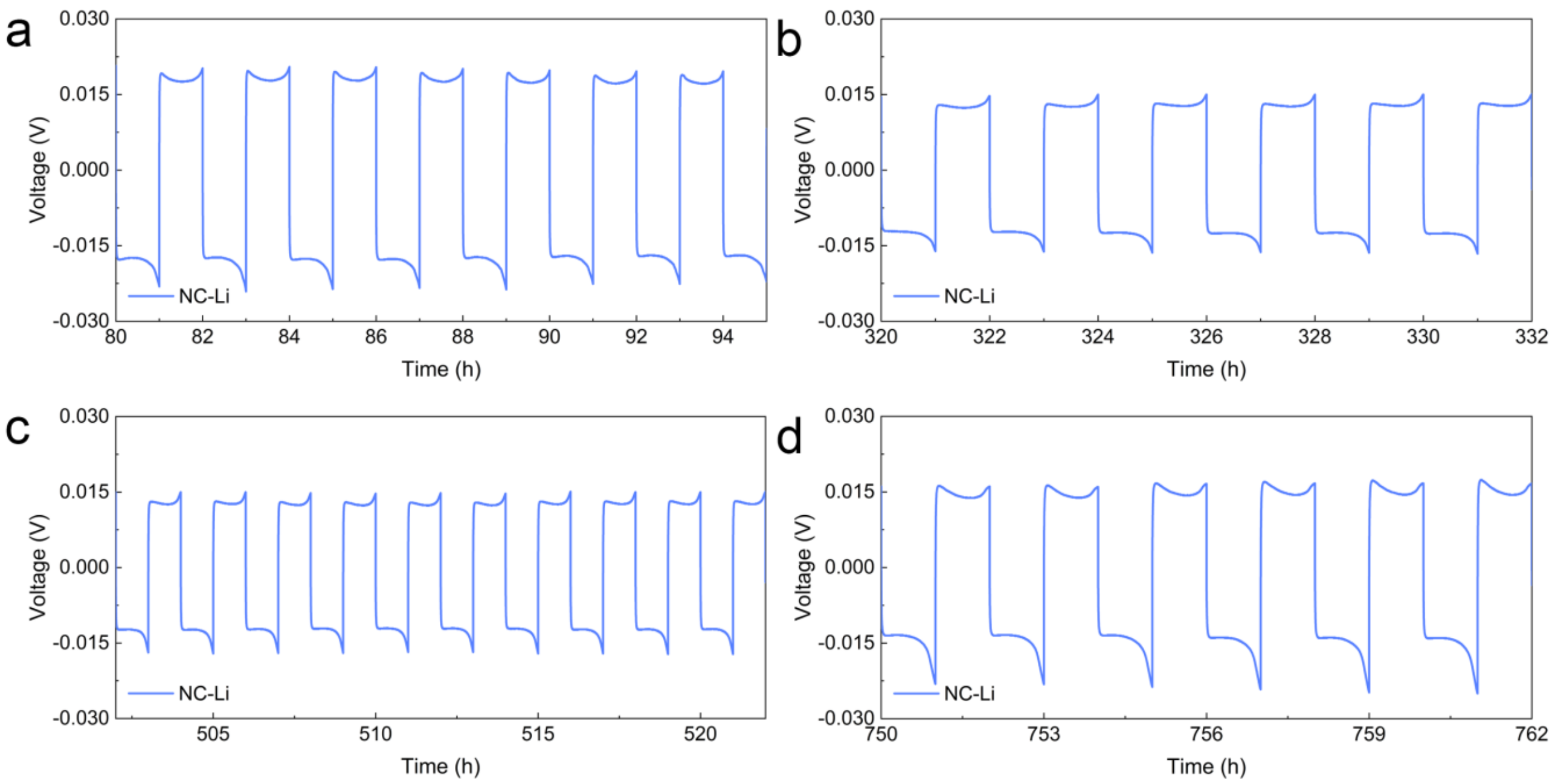

Figure S28. Enlarged voltage profiles of symmetric cell with NC-Li electrodes tested at 1 mA cm^{-2} and 1 mAh cm^{-2}.

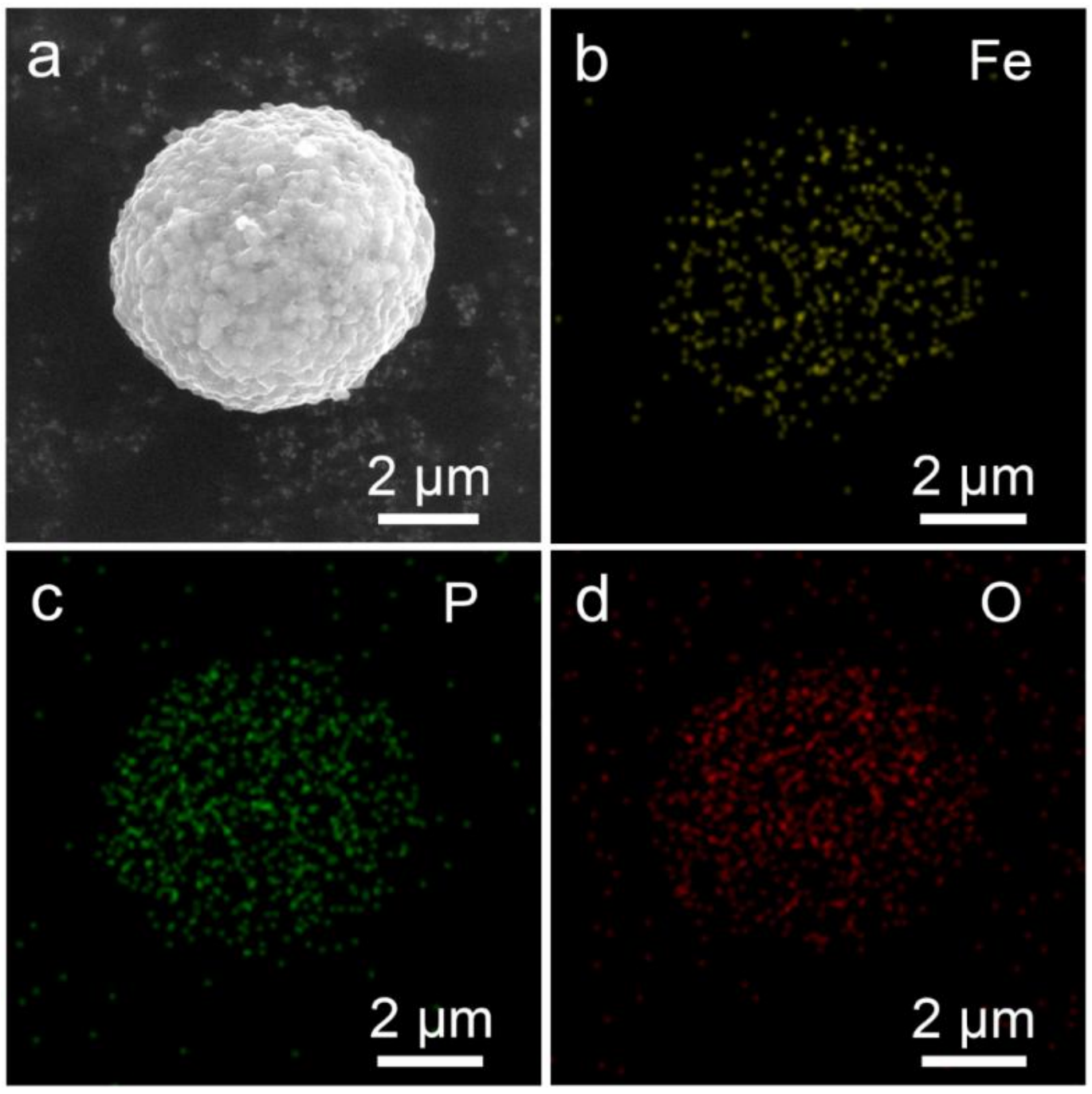

Figure S29. (a) FESEM and (b to d) EDX mapping images of LFP.

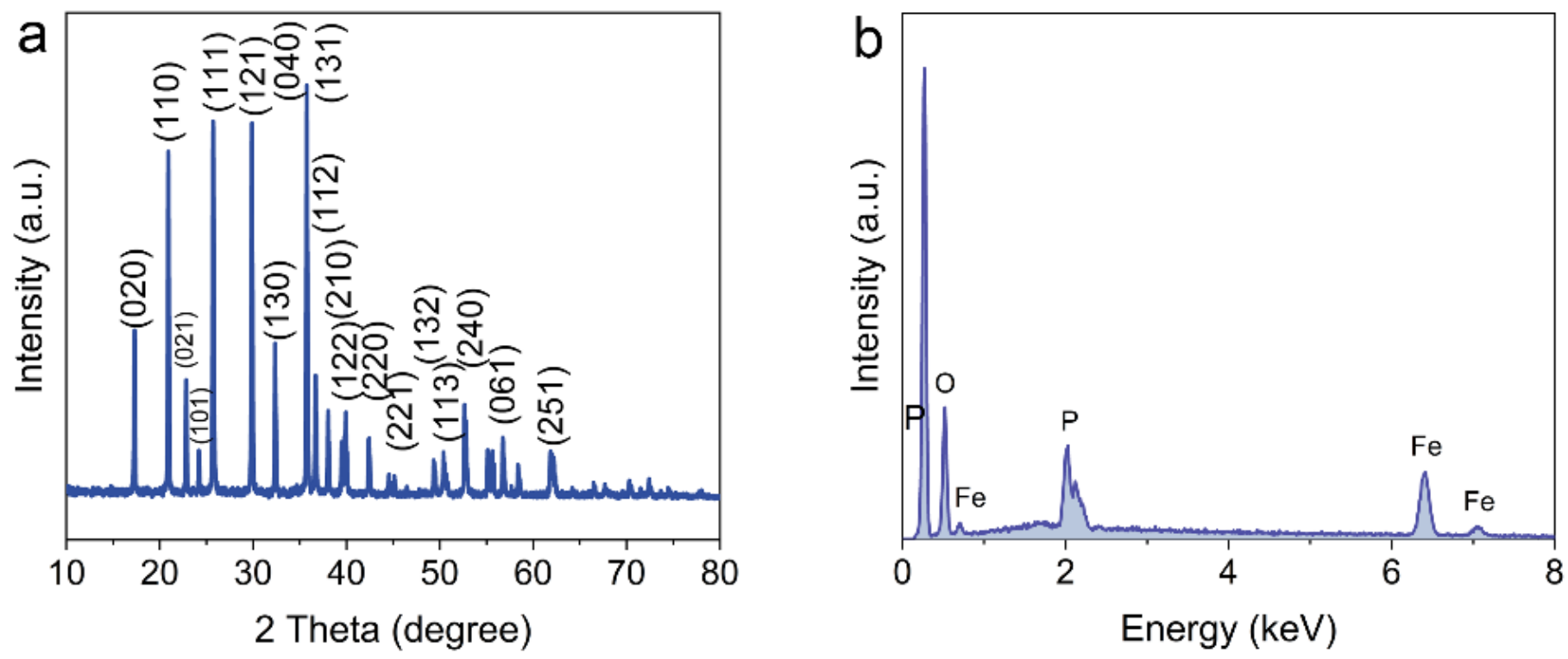

Figure S30. (a) XRD pattern and (b) EDX spectrum of LFP.

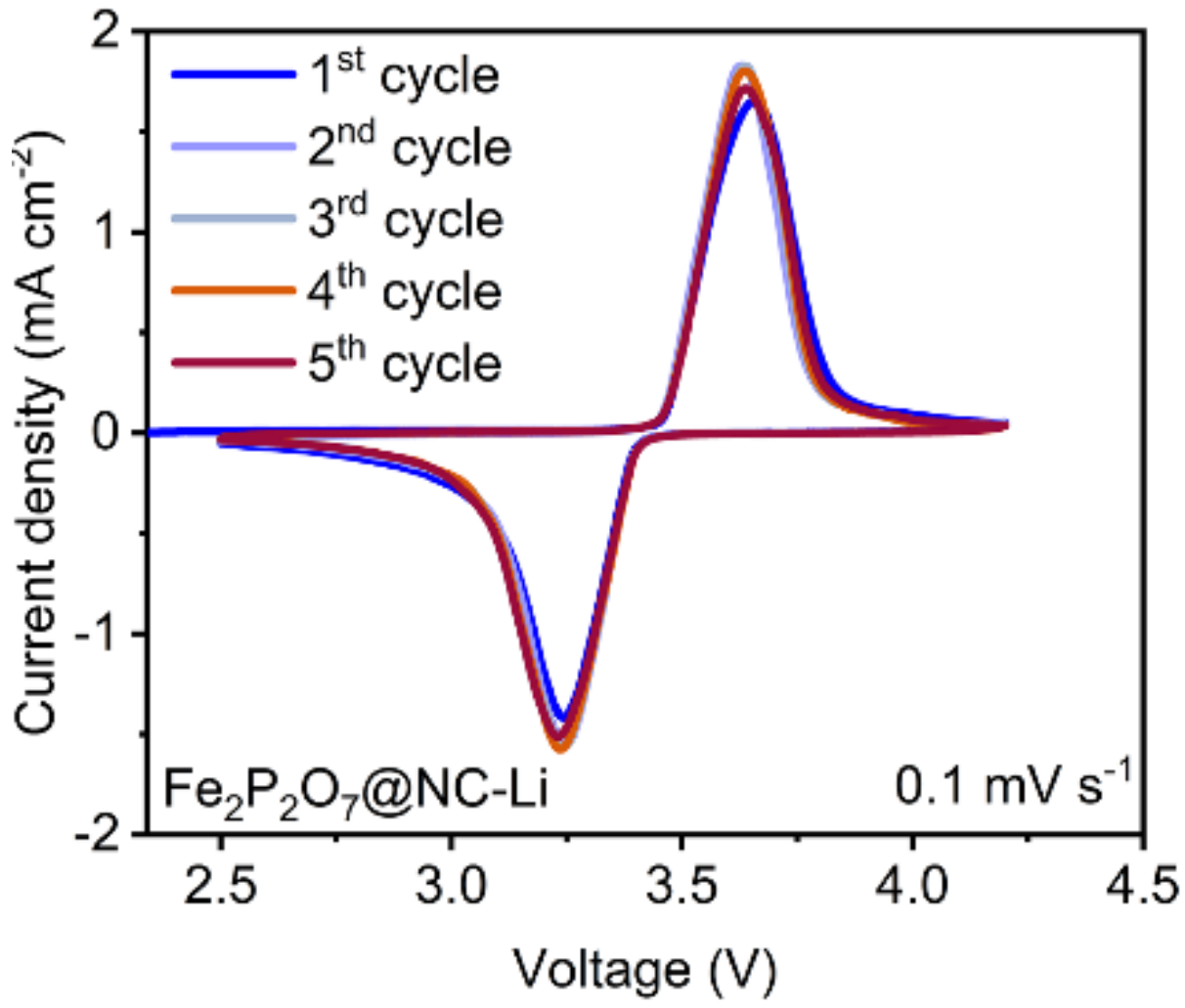

Figure S31. CV curves of $Fe_2P_2O_7$@NC-Li//LFP cell tested at a scan rate of 0.1 mV s^{-1}.

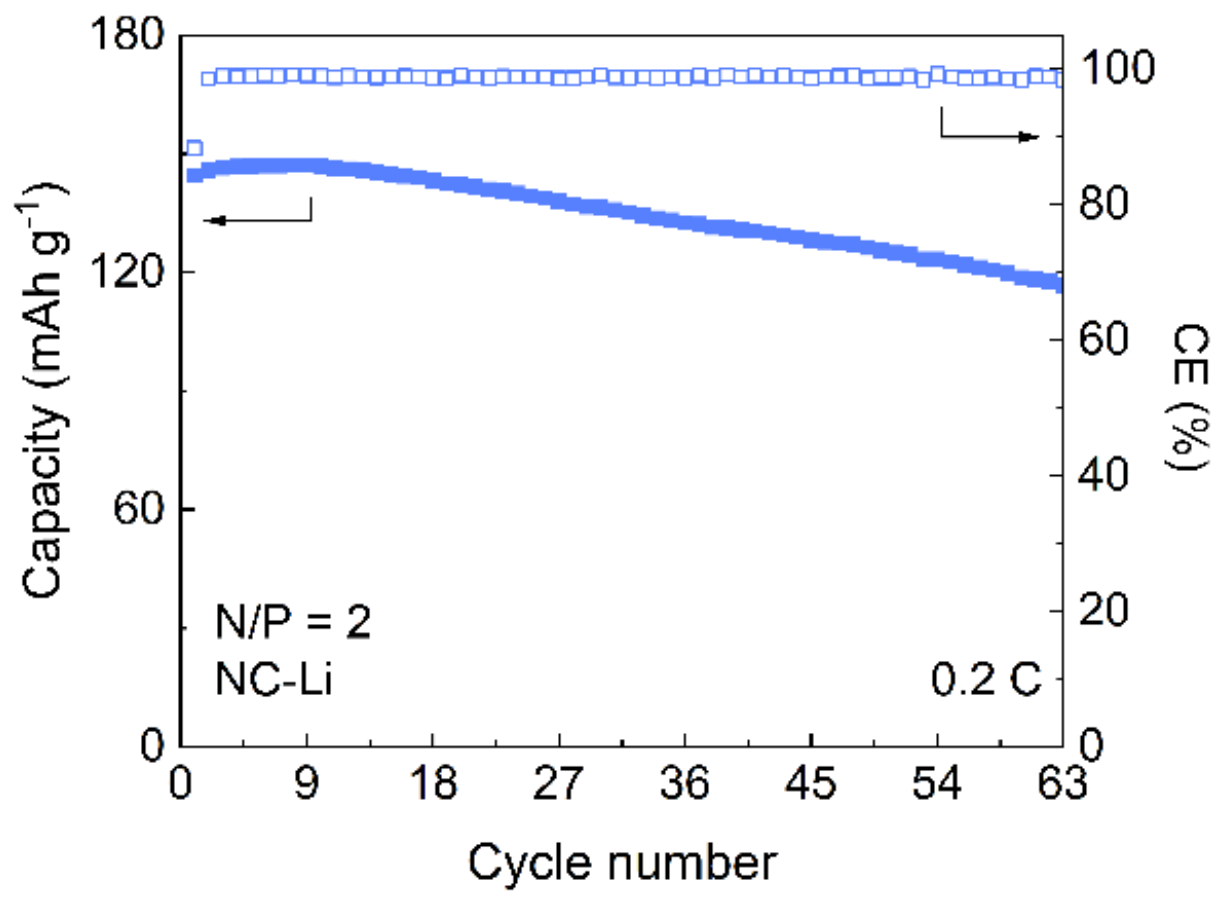

Figure S32. Cycling performance of NC-Li//LFP cell tested at 0.2 C

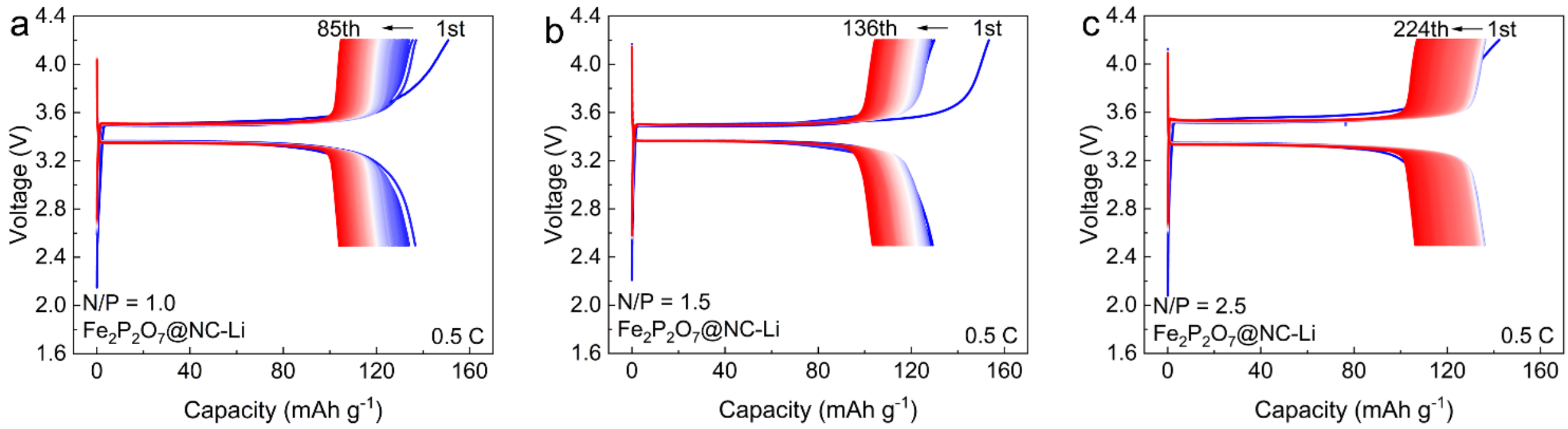

Figure S33. Charge/discharge profiles of $Fe_2P_2O_7$@NC-Li//LFP cells under different N/P capacity ratios of (a) 1.0, (b) 1.5, and (c) 2.5 at 0.5 C.

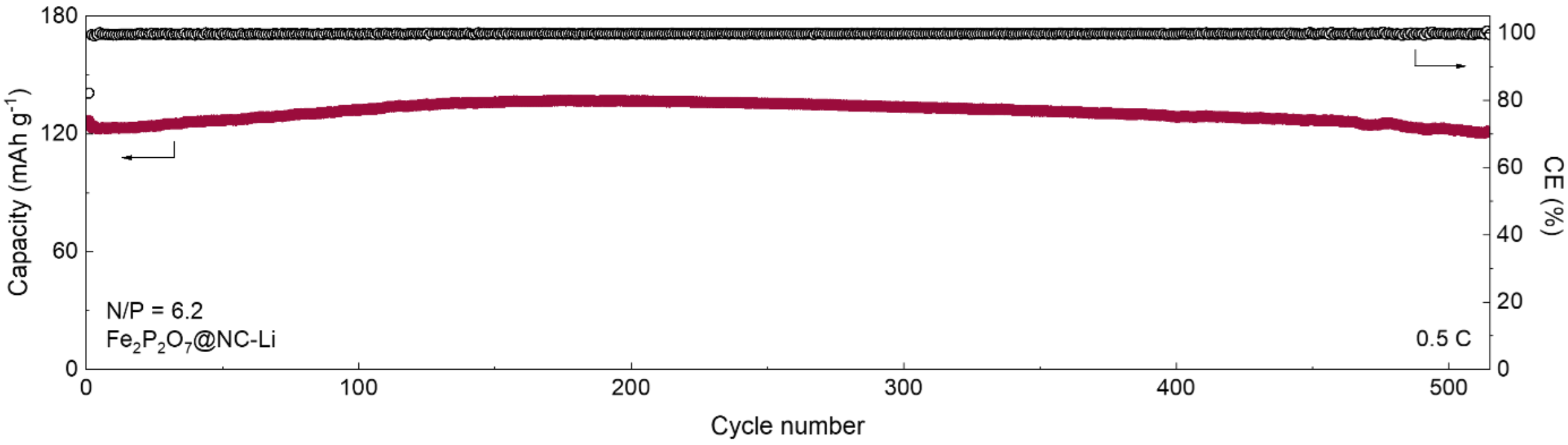

Figure S34. Cycling performance of $Fe_2P_2O_7$@NC-Li//LFP cell under a N/P capacity ratio of 6.2 at 0.5 C.

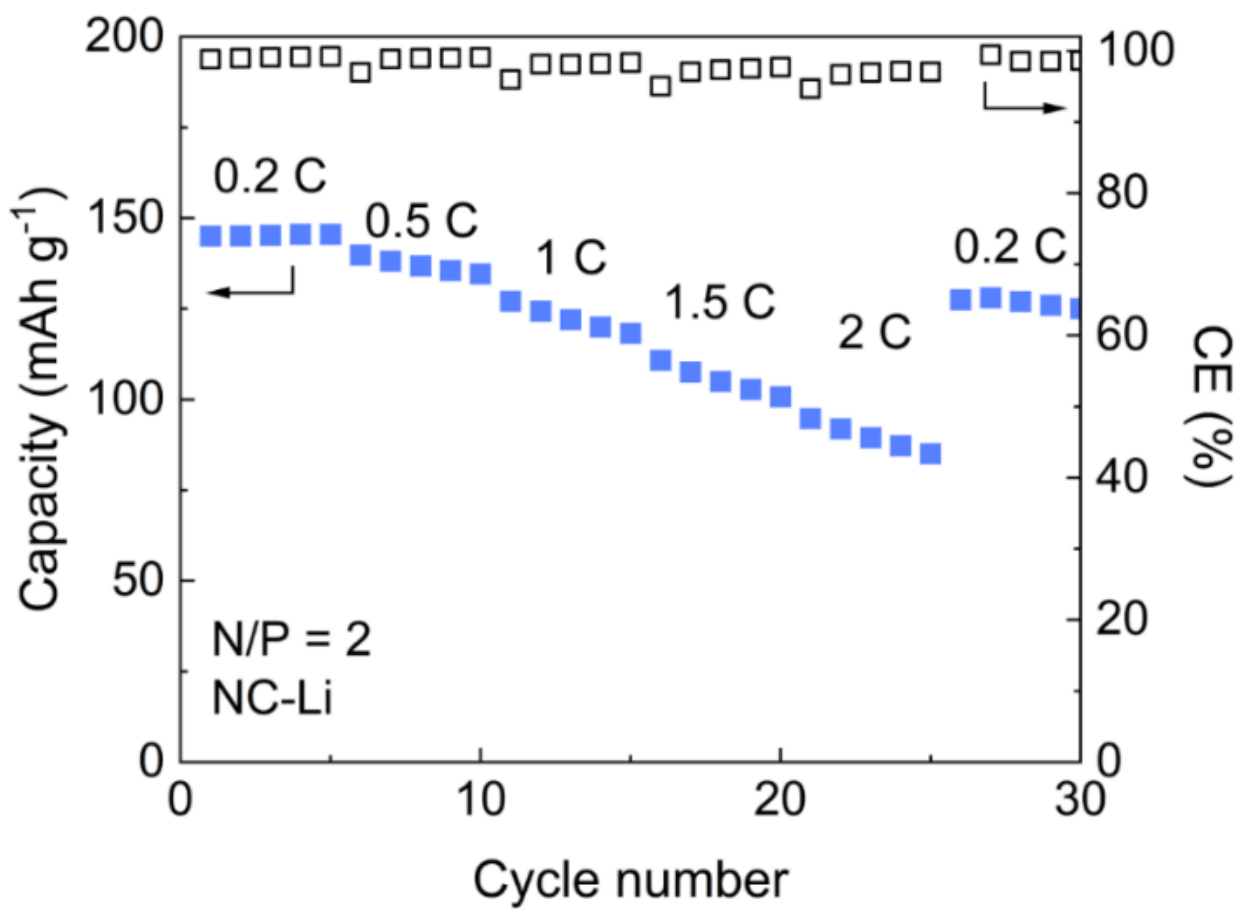

Figure S35. Rate capability of NC-Li//LFP cell tested at different current densities under a N/P ratio of 2.

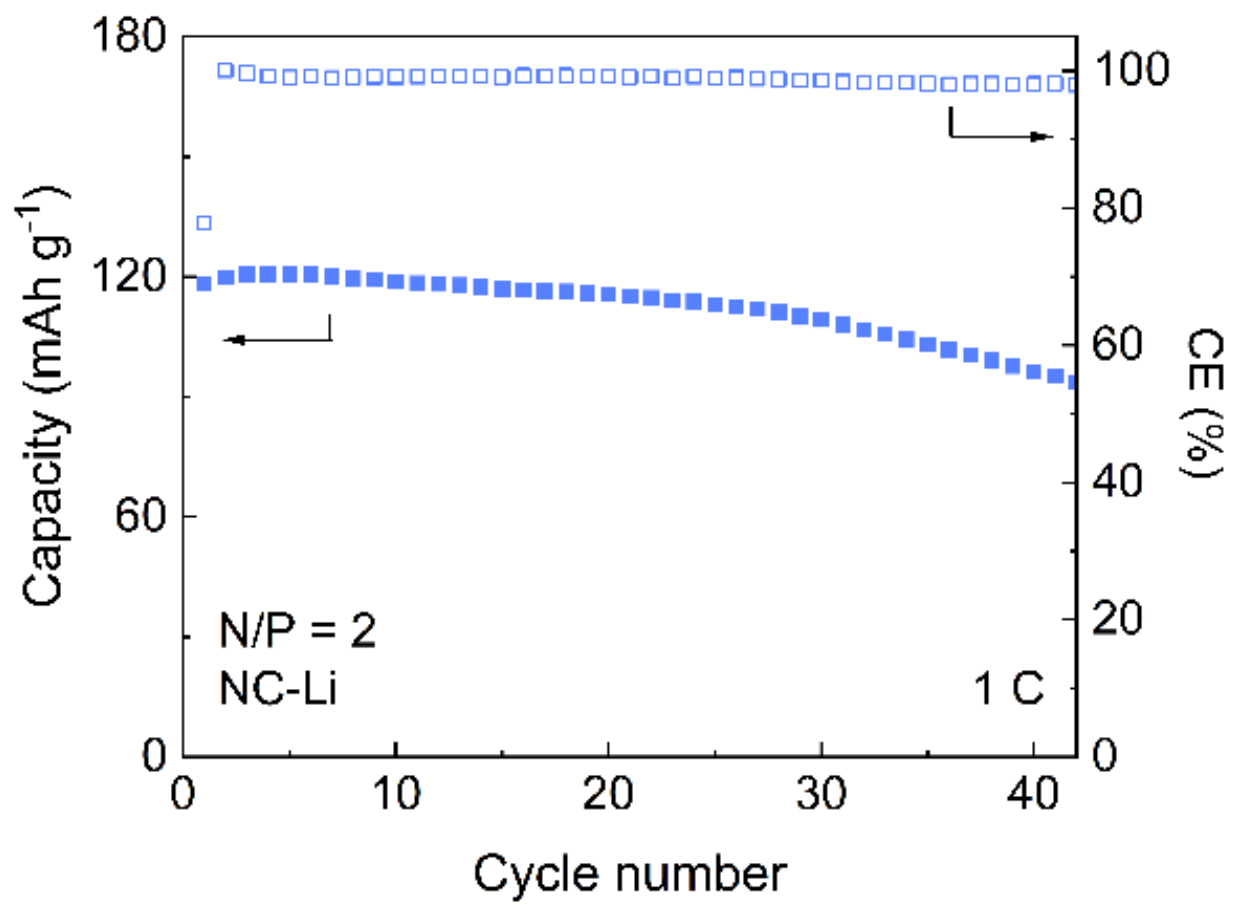

Figure S36. Cycling performance of NC-Li//LFP cell tested at 1C (N/P=2).

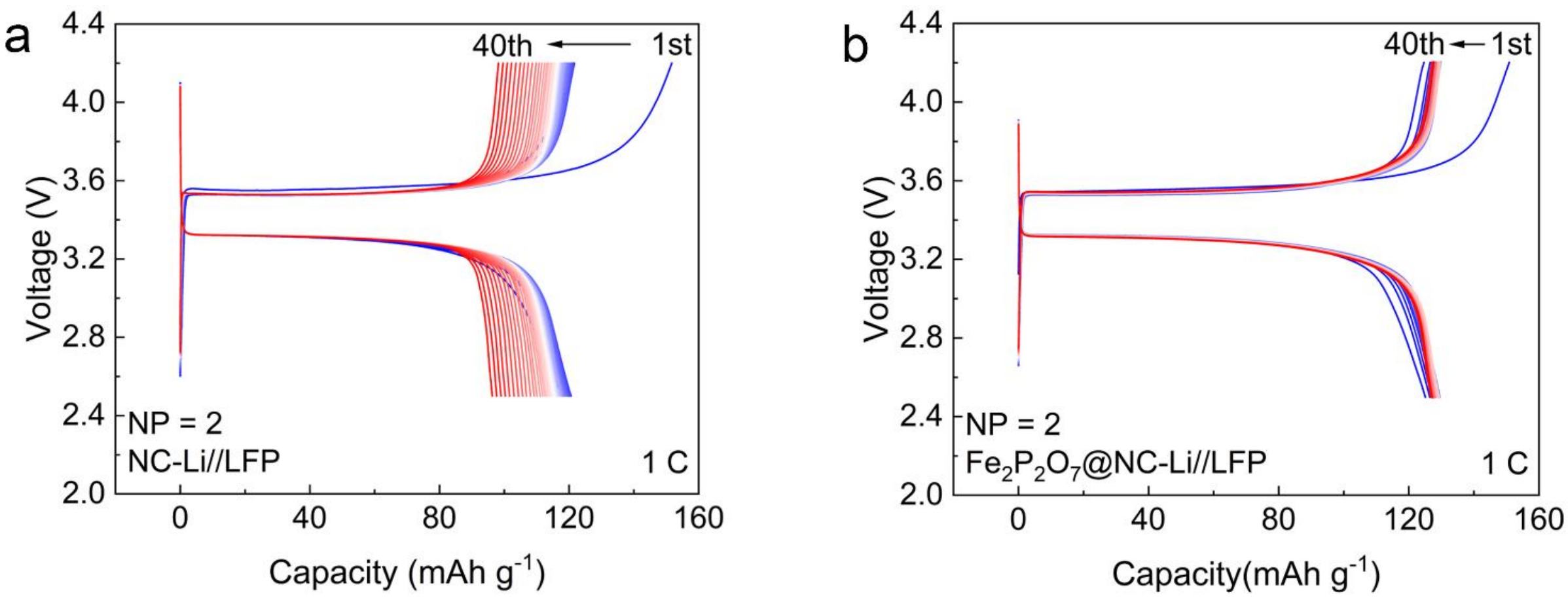

Figure S37. Comparison of charge/discharge profiles from 1st to 40th cycle of (a) NC-Li//LFP and (b) $Fe_2P_2O_7$@NC-Li//LFP cells at 1 C under a N/P capacity ratio of 2.

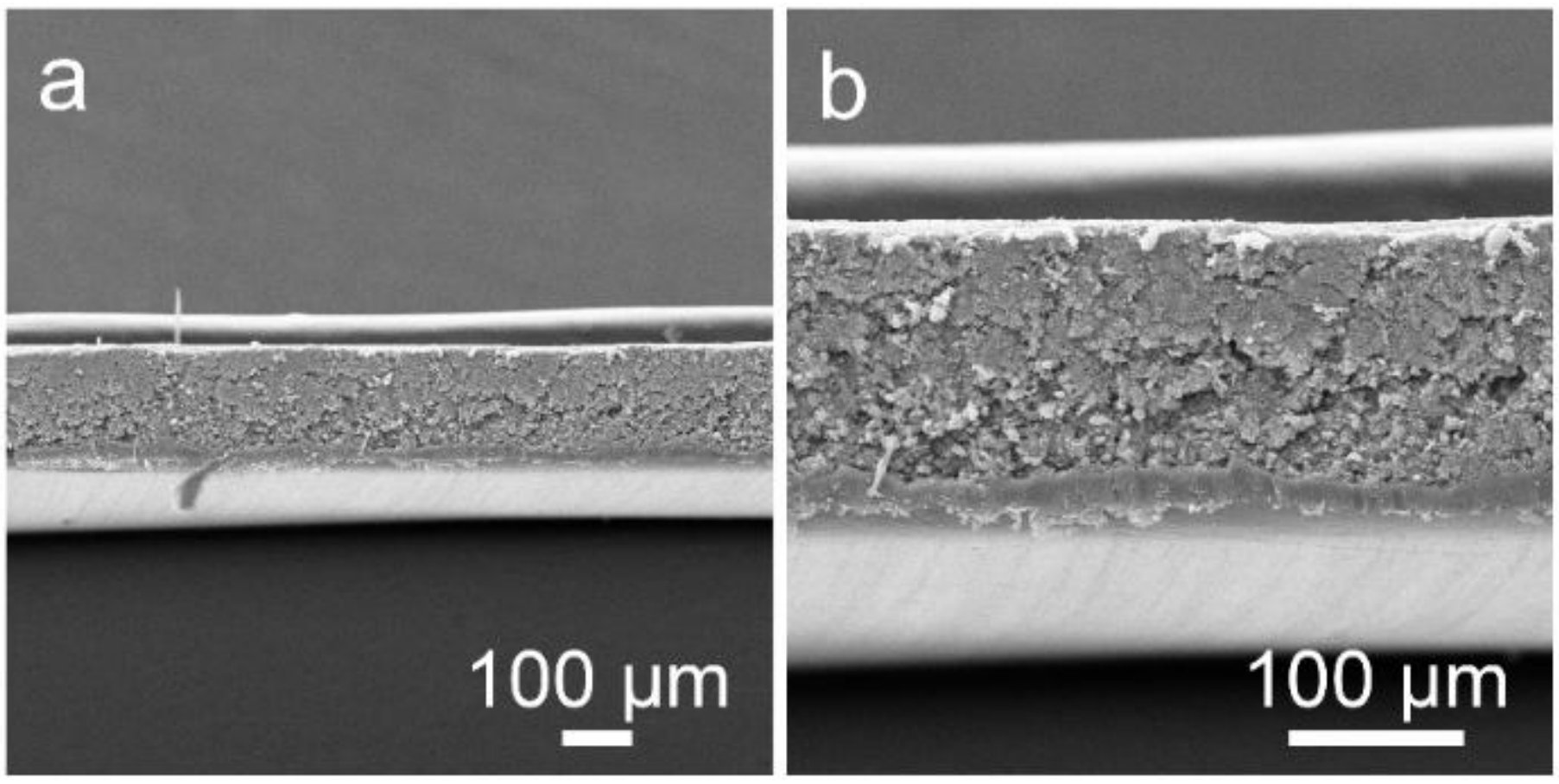

Figure S38. Cross-sectional FESEM images of LFP cathode with a high mass loading of ~18 mg cm^{-2}.

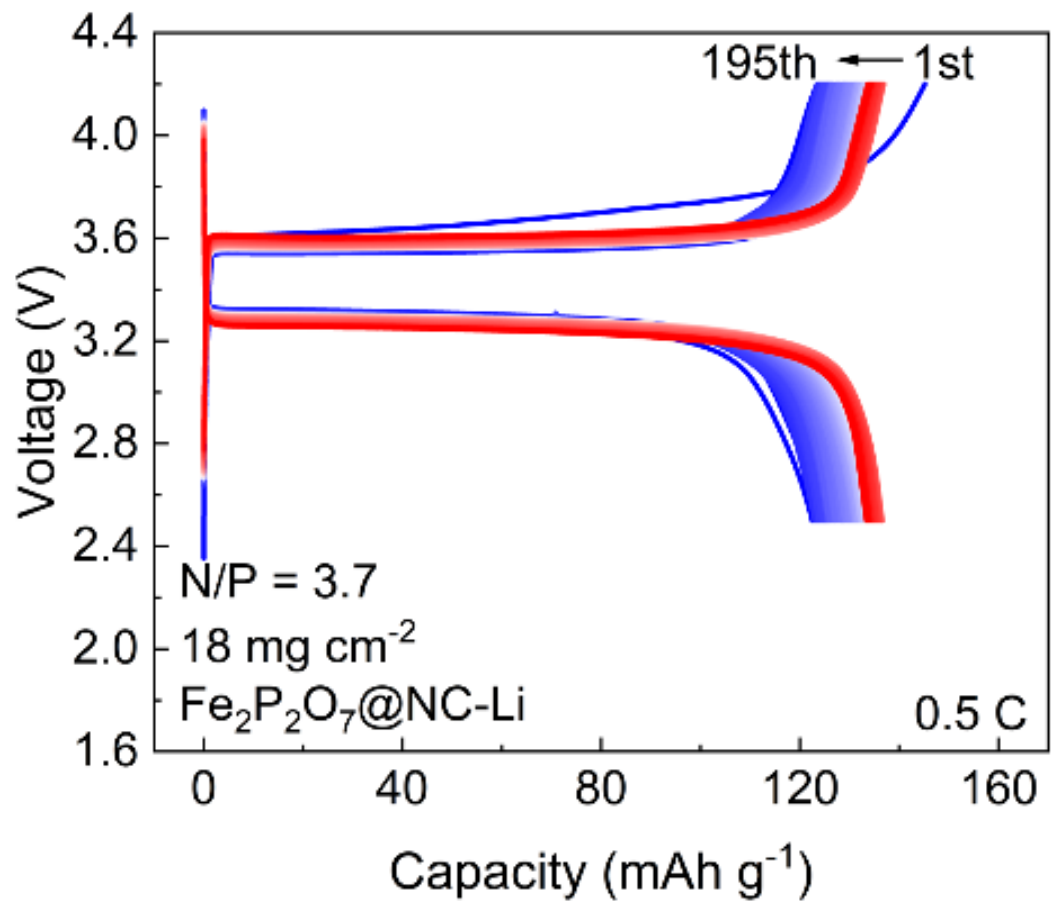

Figure S39. Charge/discharge profiles of $Fe_2P_2O_7$@NC-Li//LFP cells with a high mass loading of 18 mg cm^{-2} under a N/P capacity ratio of 3.7 at 0.5 C.

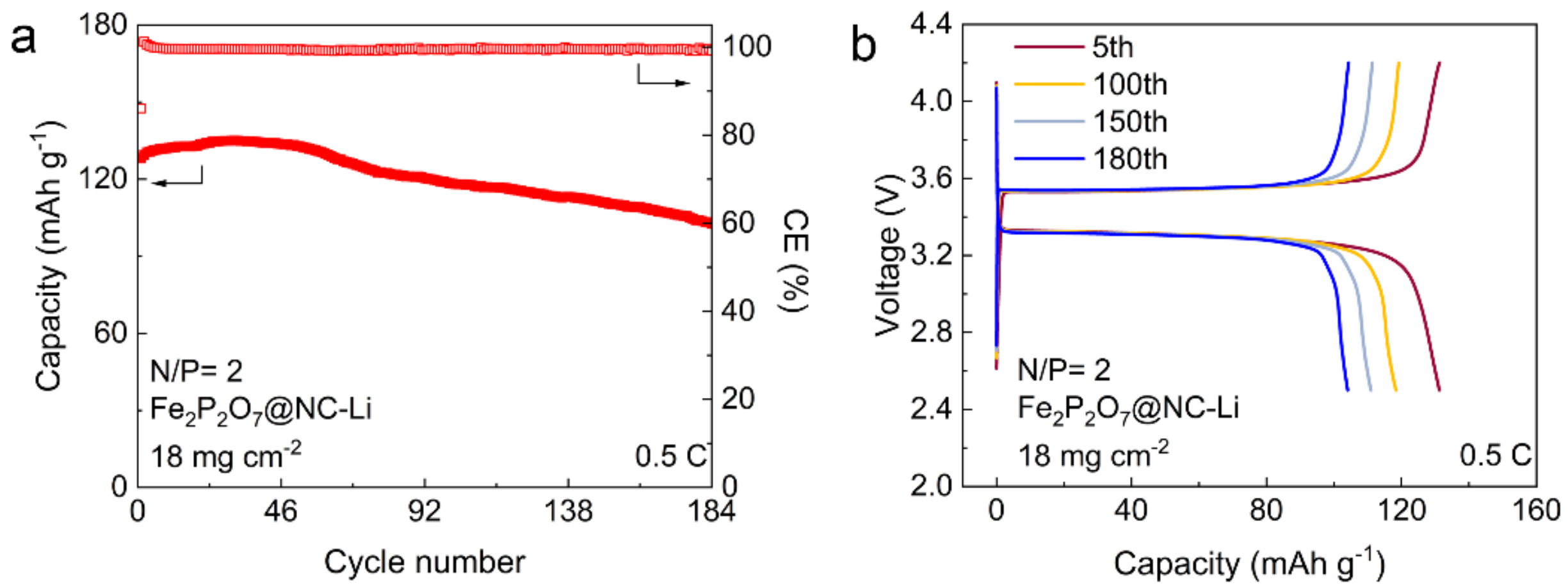

Figure S40. Cycling performance of $Fe_2P_2O_7$@NC-Li//LFP cell with a high-mass-loading cathode (18 mg cm^{-2}) at 0.5 C under a N/P capacity ratio of 2 (a) and corresponding charge-discharge profiles of different cycles (b).

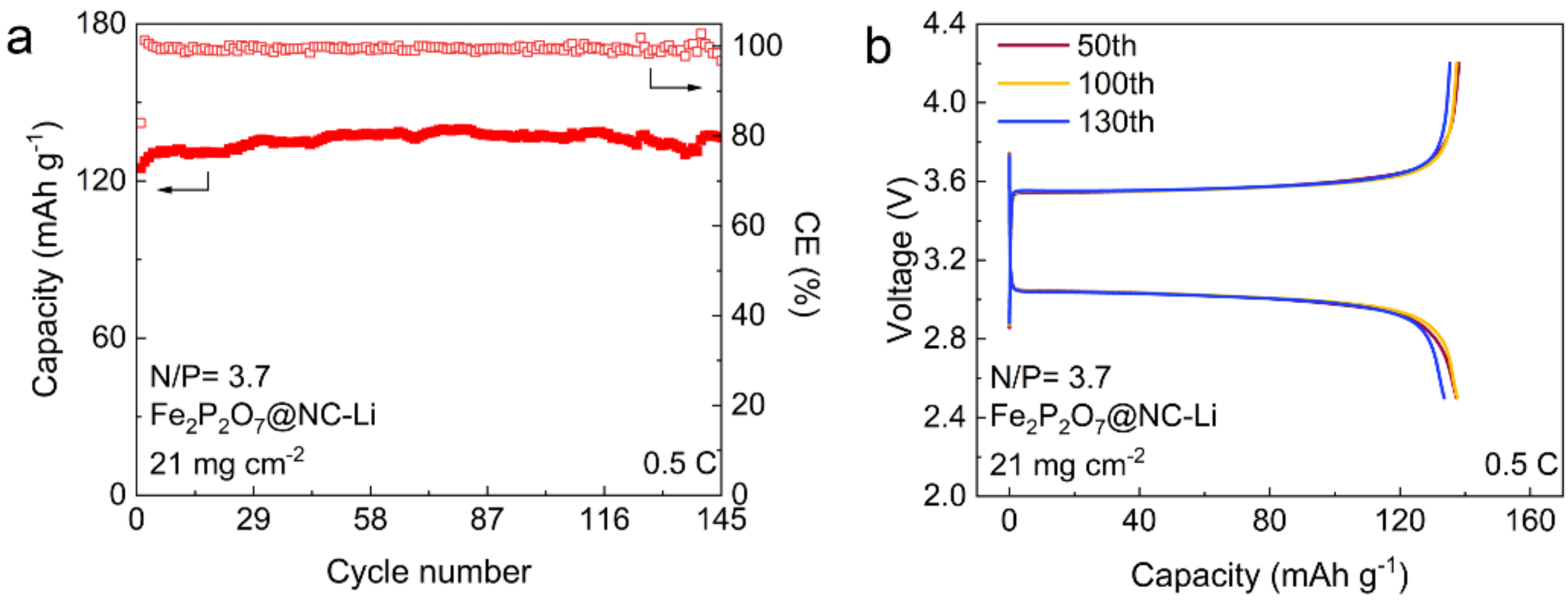

Figure S41. Cycling performance of $Fe_2P_2O_7$@NC-Li//LFP cell with a high-mass-loading cathode (21 mg cm^{-2}) at 0.5 C under a N/P capacity ratio of 3.7 (a) and corresponding charge-discharge profiles of different cycles (b).

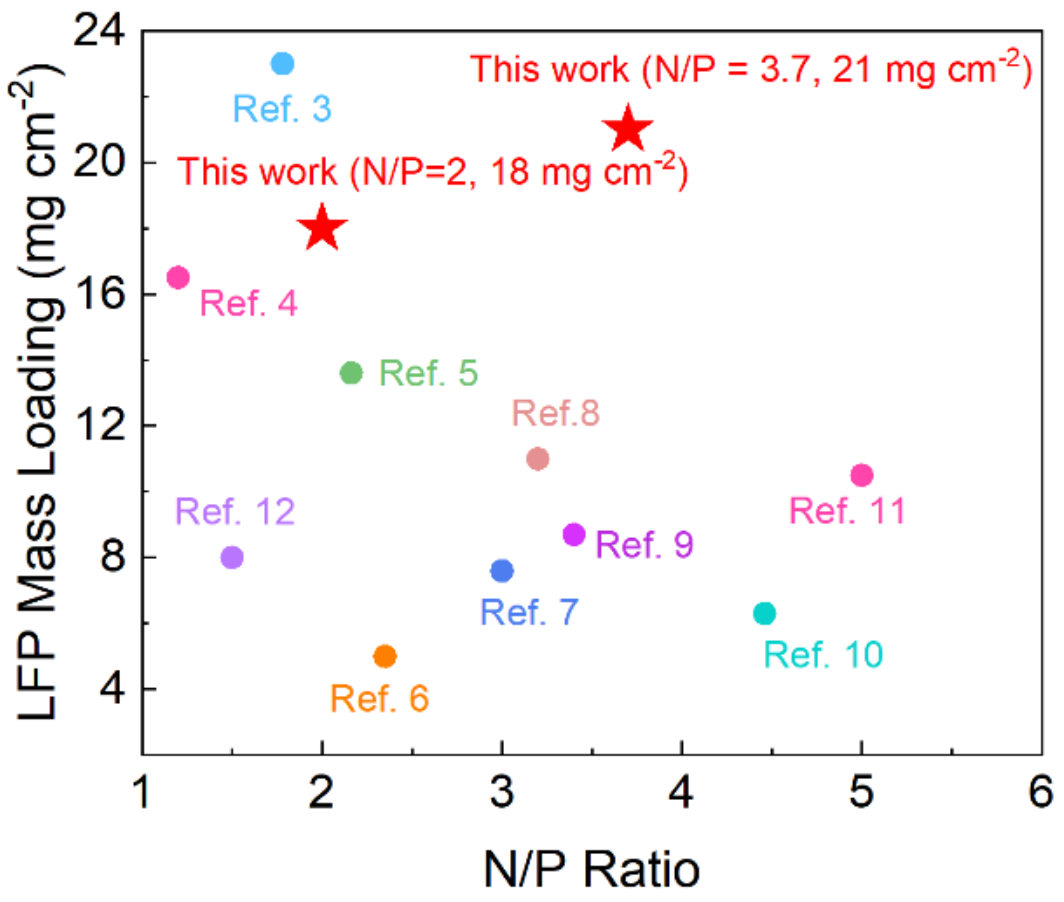

Figure S42. Comparison of the configuration of LMB full cells in this work with some other reported LMBs.

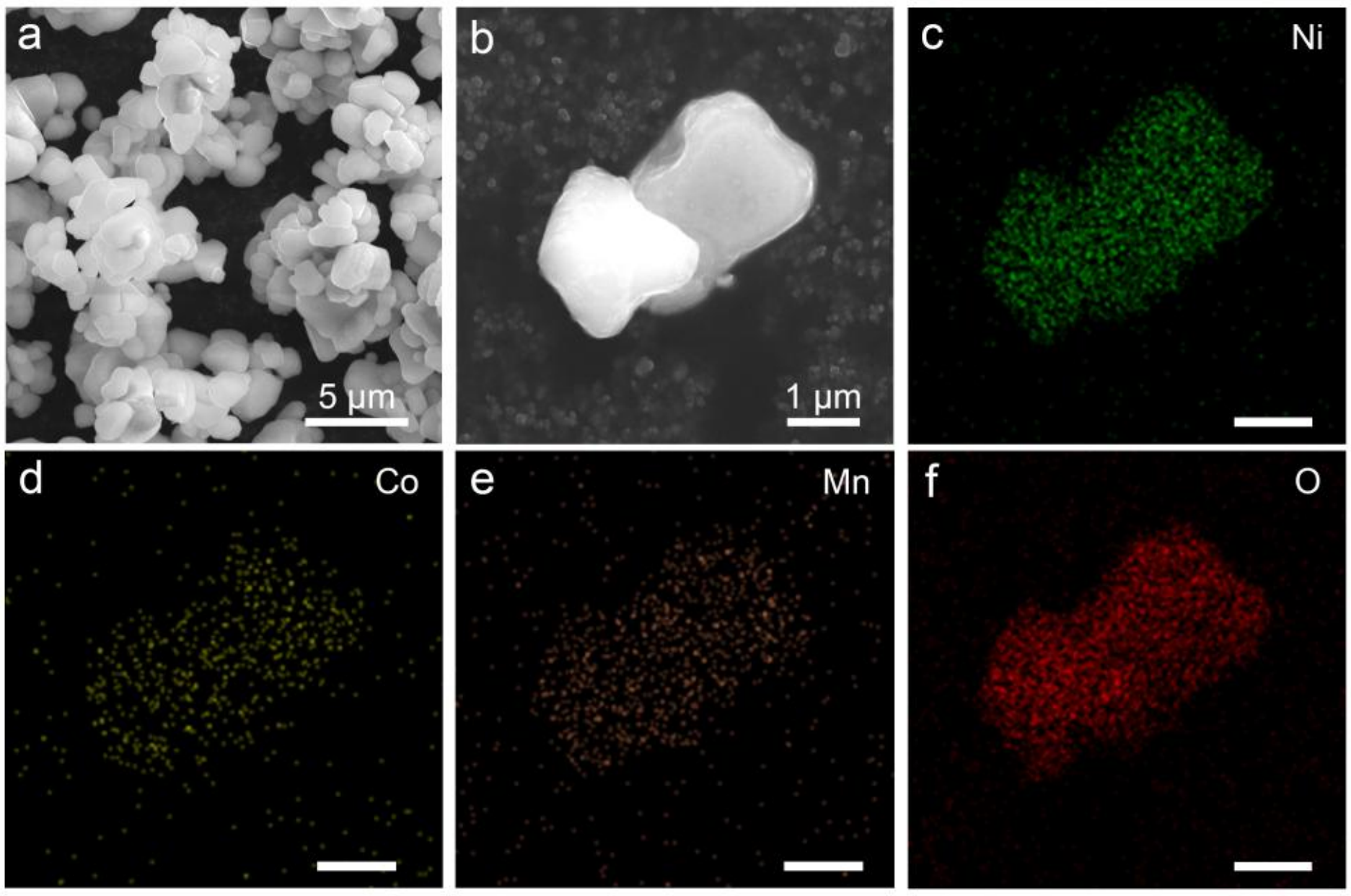

Figure S43. (a-b) FESEM and (c-f) EDX elemental mapping images of NCM811 powder. The scale bars in (c-f) are 1 μm.

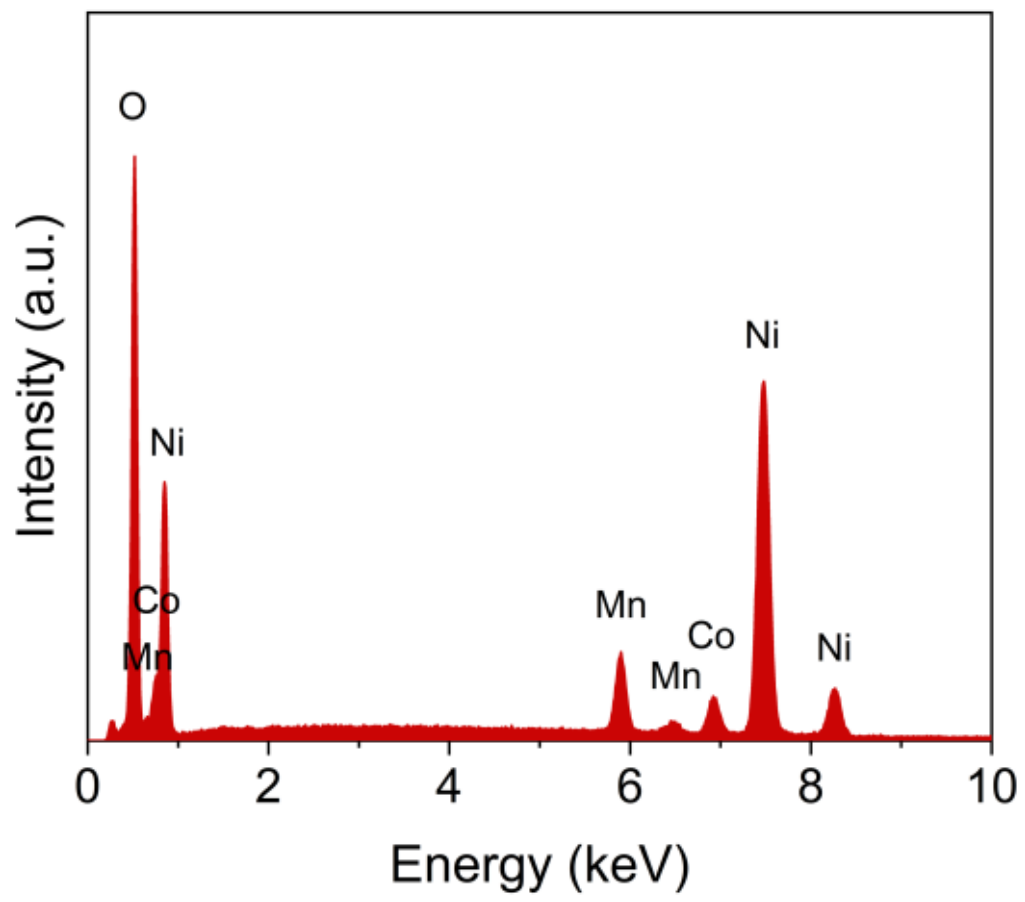

Figure S44. EDX spectrum of NCM811 powder.

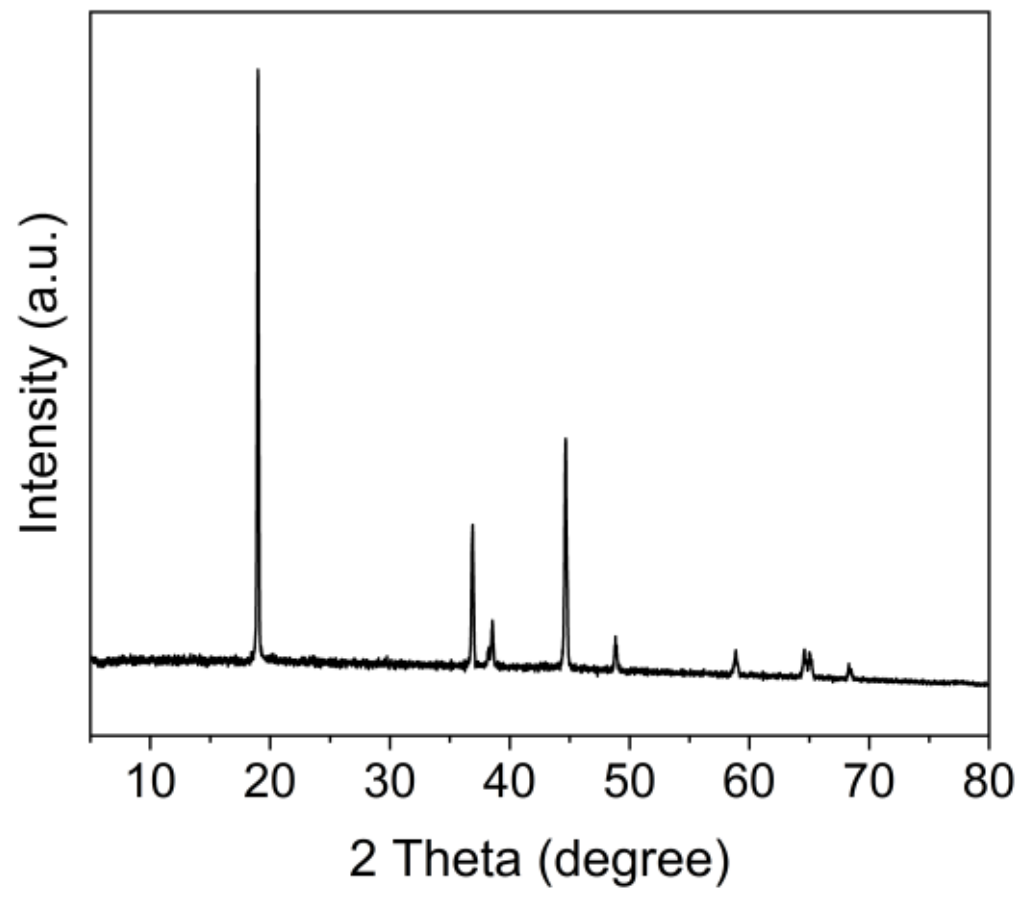

Figure S45. XRD pattern of NCM811 powder.

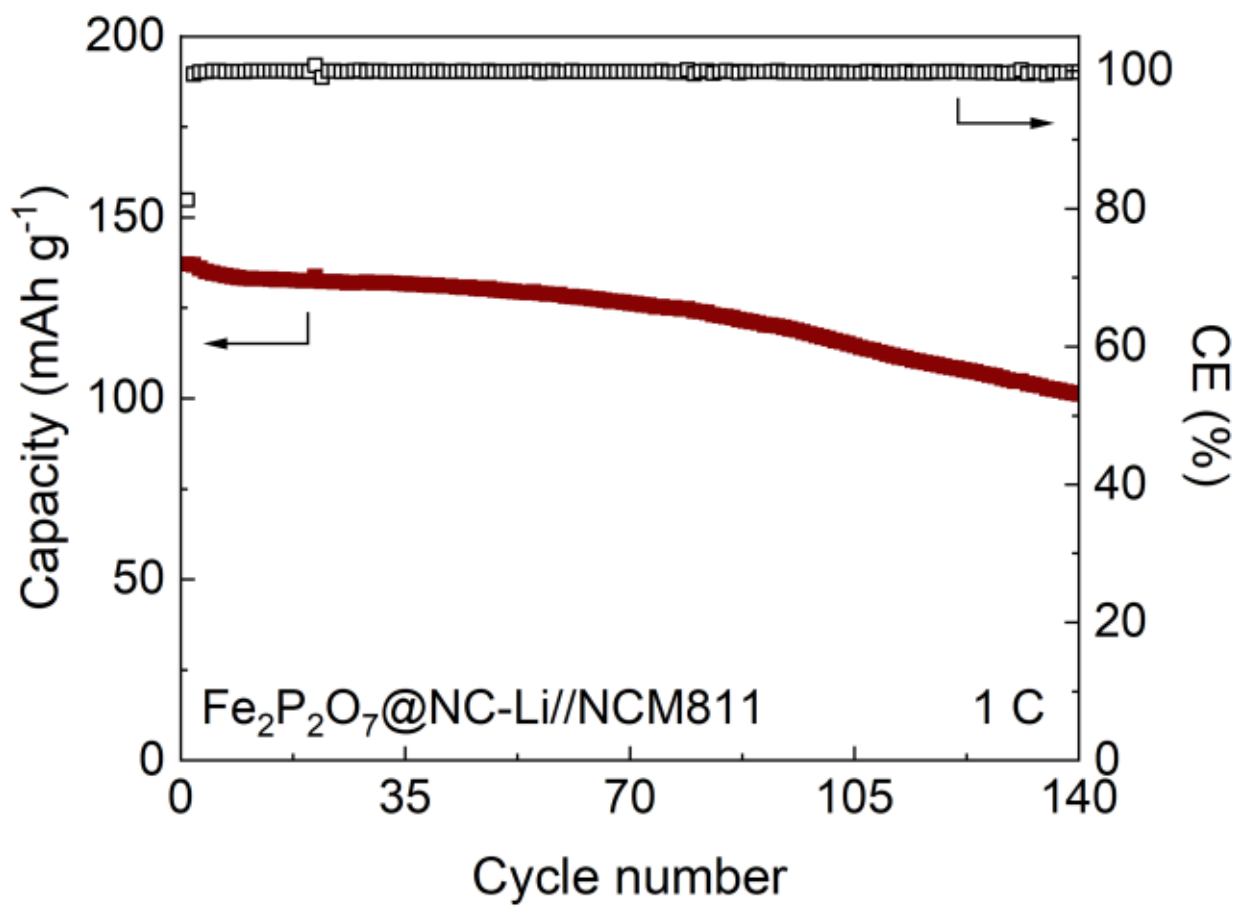

Figure S46. Cycling performance of $Fe_2P_2O_7$@NC-Li//NCM811 full cell at 1 C.

Table S1. Comparison table of cycling performance of $Fe_2P_2O_7$@NC-Li anode and other reported Li anodes in full cells with LFP cathode.

Anode	N/P ratio	Rate (C)	Mass loading (mg cm^{-2})	Electrolyte	Cycle number	Capacity retention (%)	Ref.
**$Fe_2P_2O_7$@ NC-Li**	2.5	0.5	7.46	1 M LiTFSI in DOL/ DME (v:v = 1/1) with 2 wt% $LiNO_3$	224	89.2	**This work**
	3.7		18		195	99.66	
**LZ-rGO**	1.2	0.5	4.25	1 M LiTFSI in DOL/ DME (v:v = 1/1) with 2 wt% $LiNO_3$	80	~96.5	[13]
**Co-CNF-Li**	2.63	0.2	23	1 M LiTFSI in DOL/ DME (v:v = 1/1) with 1 wt%$LiNO_3$	110	97.2	[3]
**Si-HCF-Li**	3.6	0.5	4.88	1 M LiTFSI in DOL/ DME (v:v = 1/1) with 1 wt%$LiNO_3$	400	80.7	[14]
**Li-COF**	2	0.3	23.5	1 M LiTFSI in DOL/DME (v:v = 8:2) with 10 vol% FEC	~150	91	[15]
**Ni_3S_2 NWs-NF-Li**	1.5	0.5	11.76	1 M LiTFSI in DOL/ DME (v:v = 1/1) with 1 wt%$LiNO_3$	70	95.4	[16]
**CNF-TiN-Li**	3.53	1	5	1 M LiTFSI in DOL/ DME (v:v = 1/1) with 1 wt%$LiNO_3$	250	74.9	[17]
**LS@A-Li**	-	1 C	2.85	1 M LiTFSI in DOL/ DME (v:v = 1/1)	~750	88	[18]
**TESM/Li**	3.3	1	22.4	1 M LiTFSI in DOL/ DME (v:v = 1/1) with 1 wt%$LiNO_3$	~163	93.7	[19]
**VA-CPE-Li**	-	0.2	1.5	1 M LiTFSI in DOL/ DME (v:v = 1/1)	200	92	[20]
**NF-Cu@Li**	~4.46	0.5	~6.3	4 M LiTFSI in DME	75	94.4	[10]
**LGP-Li**	~70.6	0.5	0.5	1 M LiTFSI in DOL/ DME (v:v = 1/1)	300	74.3	[21]
**rGO-S-Li**	3.92	1	3	1 M LiTFSI in DOL/ DME (v:v = 1/1) with 2 wt% $LiNO_3$	300	72	[22]

Supplementary References

[1] X. F. Lu, L. Yu, X. W. Lou, *Sci. Adv.* **2019**, 5, eaav6009.

[2] S. Wu, X. Lu, Y. Sun, H. Wang, M. A. Waseem, J. Aslam, Y. Xu, L.-P. Lv, Y. Wang, *Angew. Chem. Int. Ed.* **2025**, 64, e202506892.

[3] Y.-S. Feng, Y.-N. Li, P. Wang, Z.-P. Guo, F.-F. Cao, H. Ye, *Angew. Chem. Int. Ed.* **2023**, 62, e202310132.

[4] G. Lee, D.-Y. Han, S. Park, S. Lee, S. Choi, S. Kim, Y. Song, N.-S. Choi, H.-W. Lee, J. Moon, S. Park, *Adv. Mater.* **2026**, 38, e10919.

[5] R. Zhang, J. Jiang, J. Guo, X. Liao, J. Liu, H. Wang, J. Xu, D. Cao, P. Cheng, W. Shi, *Adv. Mater.* **2025**, 37, e07827.

[6] H. Wu, L. Wu, Y. Li, W. Dong, W. Ma, S. Li, D. Xiao, P. Huang, X. Zhang, *Angew. Chem. Int. Ed.* **2025**, 64, e202417209.

[7] W. Fu, K. Chen, F. Wang, Y. Wang, E. Wilson, V. Chandra, D. Kim, G. Yushin, *ACS Energy Lett.* **2025**, 10, 853.

[8] S. Huang, T. Li, Z. Xie, K. Li, Y. Chen, L. Mei, G.-C. Kuang, Z. Wu, Y. Zhang, L. Gu, L. Chen, *Adv. Mater.* **2026**, 38, e19442.

[9] J. Cao, Y. Shi, A. Gao, G. Du, M. Dilxat, Y. Zhang, M. Cai, G. Qian, X. Lu, F. Xie, Y. Sun, X. Lu, *Nat. Commun.* **2024**, 15, 1354.

[10] X. Li, R. Yang, L. Xia, D. Chen, J. Zhu, Y. Yan, W. Chen, D. Chen, C. Yan, T. Lei, C. Zhang, K. Cheng, Y. Hu, *Adv. Funct. Mater.* **2026**, 36, e09246.

[11] S. Huang, H. Liu, K. Li, Z. Xie, W. Li, L. Mei, L. Chen, *Nano Lett.* **2025**, 25, 17659.

[12] Y. Jeong, C. Heo, M. Kim, G. Lee, *ACS Nano* **2025**, 19, 34163.

[13] X. Zhou, S. Wang, Y. Li, Y. Yang, X. Xiao, G. Chen, *Adv. Energy Mater.* **2025**, 15, 2403640.

[14] S. Wang, J. Zhang, L. Zhang, X. Hu, X. Qin, X. Yan, Z. Wang, X. Lu, Y. Xin, F. Kang, H. Tian, B. Li, *Nano Energy* **2024**, 131, 110255.

[15] X. Wu, S. Zhang, X. Xu, F. Wen, H. Wang, H. Chen, X. Fan, N. Huang, *Angew. Chem. Int. Ed.* **2024**, 63, e202319355.

[16] J. Pan, K. Shi, H. Wu, J. Li, R. Zhang, Q. Liu, Z. Liang, *Adv. Energy Mater.* **2024**, 14,

2302862.

[17] K. Lin, X. Qin, M. Liu, X. Xu, G. Liang, J. Wu, F. Kang, G. Chen, B. Li, *Adv. Funct. Mater.* **2019**, 29, 1903229.

[18] J. Di, J.-L. Yang, H. Tian, P. Ren, Y. Deng, W. Tang, W. Yan, R. Liu, J. Ma, *Adv. Funct. Mater.* **2022**, 32, 2200474.

[19] Z. Ju, J. Nai, Y. Wang, T. Liu, J. Zheng, H. Yuan, O. Sheng, C. Jin, W. Zhang, Z. Jin, H. Tian, Y. Liu, X. Tao, *Nat. Commun.* **2020**, 11, 488.

[20] T. Deng, Q. Han, J. Liu, C. Yang, J. Wang, M. Wang, Z. Wang, B. Zhou, *Adv. Funct. Mater.* **2024**, 34, 2311952.

[21] G. Wang, Y. Mu, H. Gu, H. Li, H. Ren, L. Zeng, S. Xu, *Adv. Funct. Mater.* **2025**, 35, 2416495.

[22] Y. Li, Y. Hu, S. Wang, X. Zhou, Y. Yang, X. Xiao, G. Chen, *Nano Lett.* **2024**, 24, 672.
